# Assembly of planar chiral superlattices from achiral building blocks

**DOI:** 10.1038/s41467-022-31868-2

**Published:** 2022-07-21

**Authors:** Zhihua Cheng, Matthew R. Jones

**Affiliations:** 1 Department of Chemistry, Rice University, Houston, TX US; 2 Department of Materials Science & Nanoengineering, Rice University, Houston, TX US

**Keywords:** Nanoparticles, Self-assembly, Colloids

## Abstract

The spontaneous assembly of chiral structures from building blocks that lack chirality is fundamentally important for colloidal chemistry and has implications for the formation of advanced optical materials. Here, we find that purified achiral gold tetrahedron-shaped nanoparticles assemble into two-dimensional superlattices that exhibit planar chirality under a balance of repulsive electrostatic and attractive van der Waals and depletion forces. A model accounting for these interactions shows that the growth of planar structures is kinetically preferred over similar three-dimensional products, explaining their selective formation. Exploration and mapping of different packing symmetries demonstrates that the hexagonal chiral phase forms exclusively because of geometric constraints imposed by the presence of constituent tetrahedra with sharp tips. A formation mechanism is proposed in which the chiral phase nucleates from within a related 2D achiral phase by clockwise or counterclockwise rotation of tetrahedra about their central axis. These results lay the scientific foundation for the high-throughput assembly of planar chiral metamaterials.

## Abstract (web summary)

The formation of nanostructures with chiral symmetry often requires chiral directing agents at a smaller length scale. Here, the authors report the self-assembly of 2D chiral superlattices from achiral tetrahedron-shaped building blocks.

## Introduction

The tendency for matter to exhibit configurations with chiral symmetry has ramifications for a diverse set of scientific disciplines ranging from particle physics to the origin of life. For most chemical systems, chiral symmetry emerges at a given length scale only if at a smaller length scale there exists a chiral center that seeds growth^1–8^. More recently, micron- and nanometer-scale colloids that are achiral have demonstrated the ability to spontaneously assemble into ordered chiral superlattices^9–12^. However, these exclusively form linear helix or twisted ribbon architectures. Particularly for nanophotonics applications, in which chiral configurations have been intensely pursued as a route to metamaterials with negative refractive index^13–16^, the formation of planar 2D chiral assemblies would be advantageous in the improved structural control and increased scale compared to laborious lithographic techniques that are more commonly employed^17–21^.

The packing of tetrahedra (Td) represents a rich field constituting both mathematical theory, molecular dynamics simulations, and experimental colloidal chemistry. A complex phase space has been mapped in which numerous symmetric arrangements form depending on the degree of tip truncation, rounding of tips/edges, and the fundamental interparticle interactions driving assembly^22,23^. Mathematically, the densest packing of Td is the so-called dimer crystal (DC) with a density of ~85.63%^24^, but lattices constituting hexagonal, body centered cubic, diamond cubic, triclinic, icosahedral, decahedral, and quasicrystalline order have been simulated^11,22–26^; the only proposed structure with chiral symmetry reported thus far is a linear helix^23^.

Assembly or synthesis of chiral nanomaterials is generally accomplished through the introduction of chiral surface ligands that control the growth of helicoid facets^27–29^ or favor interactions between particles of like-handedness^4,6,30^. The formation of chiral superlattices from achiral building blocks is more unusual and requires a balance of attractive and repulsive forces. For example, nanoparticles with conflicting van der Waals and dipole interactions or dumbbell nanorods with attractive centers and repulsive ends have been shown to form helix or twisted ribbon morphologies^11,12,31,32^. Numerous reports have investigated the assembly of Td-shaped semiconductor nanocrystals^8,33,34^, confirming several of the mathematically-proposed structures. Both chiral helix and twisted ribbon structures have been observed to assemble from achiral tetrahedra^8,11^. However, the assembly of plasmonic Td particles and the ability for Td to form 2D chiral superlattices have yet to be examined.

In this work, we demonstrate the formation of chiral hexagonal superlattices that are one unit cell in thickness, assembled from achiral Td building blocks. This is enabled by a purification strategy that allows for particle samples with sharp tips in >95% shape yield with <2% size variation. Although in three dimensions these materials are not intrinsically chiral, we observe that they grow from and are confined to substrates, preventing their free rotation and resulting in what is known as planar chirality^17–21^. Furthermore, we find that lateral growth processes in these superlattices are kinetically-enhanced compared to mechanisms that result in out-of-plane growth, preserving their planarity and preventing the formation of a 3D crystal. The nanoparticle-based superlattices adopt equal number of both chiral enantiomers but generate domains that are large enough (>1–2 µm) such that a single handedness can be measured and manipulated. Comparison of the energetic stability of several related 2D packings of Td allows for a proposed formation mechanism in which sharp-tipped particles are required for the chiral phase to nucleate from within the achiral phase over the course of the assembly process. These findings lay the fundamental groundwork for the scalable formation of chiral plasmonic planar metamaterials.

## Results

### Purification and assembly of gold tetrahedra

**Fig. 1:**
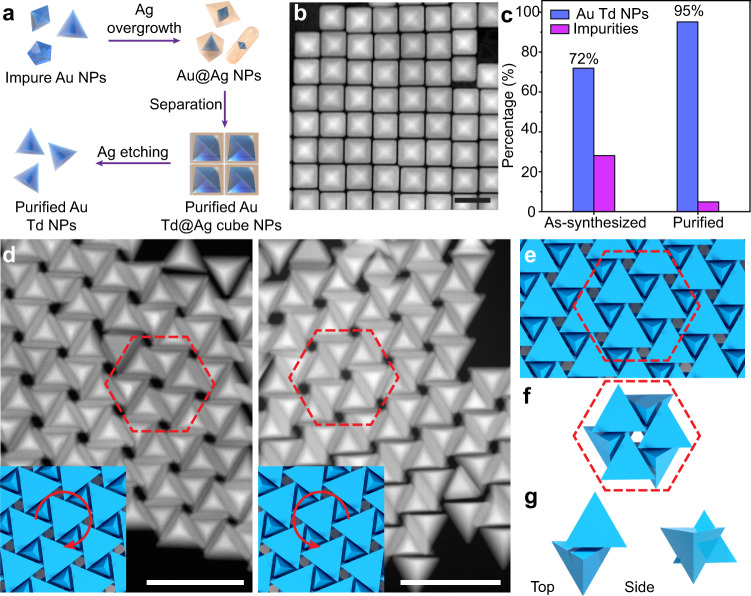
Purification and assembly of Au tetrahedra. **a** Schematic of purification methodology in which Ag shells are grown around as-synthesized samples containing a mixture of particle shapes. Small differences in the internal twinning of Au cores are accentuated in Ag shells, allowing for selective precipitation and separation of a desired morphology, e.g. tetrahedra (Td). **b** Purified Au Td@Ag cube particles. **c** Analysis of the distribution of nanoparticle shape products from as-synthesized (*n* = 7,612) and purified samples (*n* = 10,259). **d** Left and right-handed planar chiral hexagonal superlattices of tetrahedra with insets indicating orientation. **e** Schematic of the 2D chiral hexagonal phase, **f** hexagonal repeat unit, and **g** top and side views of Td dimers that constitute the structure. Scale bars: **b** 50 nm; **d** 100 nm.

The synthesis of Td-shaped gold particles is fundamentally challenging because the underlying FCC lattice falls within the *O*_*h*_ point group, thus requiring a symmetry breaking mechanism to grow into particles with *T*_*d*_ symmetry^35^. As a result, previously-reported syntheses of gold Td^36,37^ generate relatively uniform particles but with a population of impurity shapes that compete with and hinder particle crystallization (Supplementary Figs. 5 and 6). To address this, we developed a purification strategy based on the overgrowth of Ag around the products of Td nanoparticle syntheses, which acts to exaggerate the small differences in particle shape that would ordinarily make impurities difficult to separate (Fig. 1a). The resulting population of particles consist of Au Td cores surrounded by Ag cube shells, Au pentagonally twinned decahedra surrounded by Ag rod shells, and Au bitetrahedra surrounded by Ag right bipyramids (Fig. 1b, Supplementary Fig. 7). These products can be separated from one another by selective precipitation (see SI, Supplementary Figs. 8 and 9), after which the Ag shell can be etched to yield pure solutions of the original Au core particle (Fig. 1a). Analysis of over 17,000 particles shows that as-synthesized samples contain approximately 72% Td but purified samples contain approximately 95% Td (Fig. 1c, Supplementary Figs. 10, 11, Supplementary Table 1) with a size uniformity of 1.72% (Supplementary Fig. 12, Supplementary Table 2). Assembly of these particles by slow evaporation of the solvent (Supplementary Fig. 13) results in disordered structures (Supplementary Fig. 14) for the as-synthesized samples but chiral hexagonally-packed 2D superlattices for the purified samples (Fig. 1d and Supplementary Fig. 15).

**Fig. 2:**
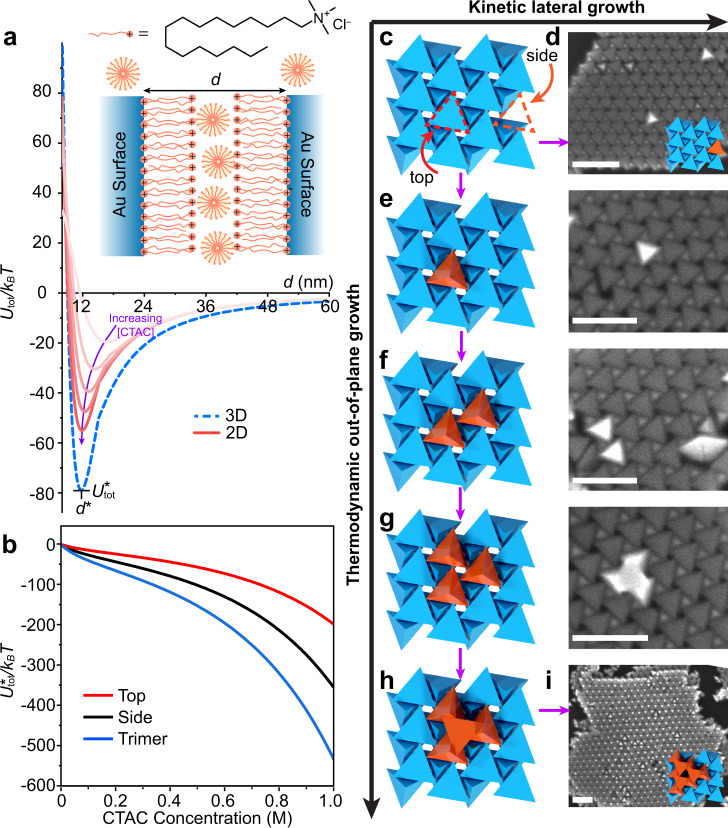
Analysis of growth modes for chiral hexagonal superlattices. **a** Net pairwise interaction potentials for 2D and 3D chiral superlattices built from tetrahedron (Td)-shaped particles. Inset illustrates the structure of hexadecyltrimethylammonium chloride (CTAC) and its ability to form both solution-phase micelles and positively charged bilayers on Au surfaces separated by a distance, *d*. The x and y positions of the potential minimum as defined as d^*^ and *U*_tot_^*^, respectively. **b** Calculation of attachment energies for a single Td particle to top **c**, side **c**, and trimer **g** positions on a superlattice. **c**, **d** Illustration and SEM image of Td attachment at favorable side positions driving lateral (2D) growth. **c**, **e**–**h** Illustration and SEM images of sequential Td attachment at unfavorable monomer **e**, dimer **f**, and trimer **g** top positions before a favorable tetramer **h** can form, allowing for out-of-plane (3D) growth. **i** SEM image of the 3D analogue of planar chiral hexagonal superlattices generated via assembly under thermodynamic conditions. All scale bars are 100 nm.

To understand the formation of hexagonally-packed 2D chiral superlattices, we developed a simple model for the interparticle potentials influencing the assembly of gold tetrahedra capped with hexadecyltrimethylammonium chloride (CTAC) ligands based on the well-developed framework of DLVO theory (Fig. 2a, inset, see methods section)^38^. The formation of a positively charged CTAC bilayer on the particle surface is responsible for repulsive electrostatic interactions^39^, while depletion forces mediated by surfactant micelles in solution and van der Waals (vdW) forces are responsible for attractive interactions^40,41^. Td particles in aqueous suspension are assembled experimentally by slowly evaporating a droplet placed on a rigid substrate (e.g., Si) in a humid environment (Supplementary Fig. 13), followed by removal of precipitated salts by chloroform before electron microscopy imaging (see methods). Consequently, the CTAC concentration slowly increases over time, resulting in an increase in the attractive depletion forces (via increasing CTAC micelle concentration) and a reduction in the repulsive electrostatic forces (via increased charge screening from electrolytes), both of which result in a gradually strengthening interparticle potential that favors crystallization. These conditions also result in interactions that are short-ranged (5–10 nm or less) relative to the particle size (66.3 nm) for the vast majority of CTAC concentrations under which assembly is taking place (Supplementary Figs. 16–18), necessitating calculation of only nearest-neighbor particle interactions in order to capture the energetic stability of different Td superlattices. We use this model to examine how the equilibrium interparticle surface-surface spacing (d^*^) and interaction potential well depth (*U*_tot_^*^) change with CTAC concentration (Fig. 2a, Supplementary Fig. 16), taking into account the changing ionic strength, counterion dissociation, and size of CTA^+^ micelles (see methods for details).

The conditions described above allow for the calculated interaction potentials (*U*_tot_^*^) to be interpreted as a measure of the thermodynamic stability of different Td superlattices, consistent with numerous literature reports and several foundational works in statistical mechanics (see methods for details)^40,42–46^. It should be noted that this approach ignores entropic contributions to the free energy, an assumption that is reasonable since the reduction in translational or rotational freedom in taking particles from a gas-like to a solid-like state represents the dominant entropic contribution to the superlattice formation energy and will therefore be similar regardless of the specific configuration of particles in the final structure. It will also be shown that despite this approximation, the theoretical predictions show excellent agreement with our experimental findings (*vide infra*), further corroborating the validity of the method and suggesting that a more detailed treatment (e.g., using Monte Carlo methods) can be the subject of future work. While this model does not provide quantitative predictions for specific Td superlattice parameters, it does allow for a qualitative comparison of the energies of conceivable superlattice structures such that relative thermodynamic stability and the likelihood of different assembly pathways can be evaluated with a high degree of confidence. In general, this framework predicts that particle configurations which maximize the face-to-face contact area between aligned, parallel facets of neighboring particles tend to be most favored as they maximize attractive depletion interactions (Supplementary Fig. 14)^47,48^. The structures optimized by these and related directional entropic forces are not necessarily the same predicted by maximum dense packing^49^.

### Driving forces for two-dimensional growth

To understand the origin of the observed preference for two-dimensional growth, we used our model to probe the energetic stability of the chiral hexagonal lattice. Calculations indicate that an analogous 3D version of the structure is indeed more thermodynamically favorable than the 2D structure generated experimentally since it always possesses a greater facet overlap area per particle, all else being equal (Fig. 2a). This finding is important since the chirality of the material is inherently linked to the fact that it is planar, i.e., once 3D growth occurs, the structure ceases to be chiral, even when immobilized on a substrate, because alternating layers have opposing handedness. We next examined the attachment preferences for a single Td particle to different positions on an existing 2D chiral hexagonal lattice (Fig. 2b, c). Whereas binding of a Td to a top position on the lattice (favoring 3D growth) results in a single nanoparticle neighbor, binding to a side position (favoring 2D growth) results in two nanoparticle neighbors and a more favorable net interaction strength (Fig. 2b–d). For out-of-plane (3D) growth to occur, three individual Td particles must all bind to adjacent top positions, which are individually less favorable, after which a fourth particle can insert tip-down into the trimer, forming a stable island (Fig. 2e–h, Supplementary Fig. 19). Since it requires three unfavorable coordination steps to allow the equilibrium 3D lattice to form but only one favorable coordination step to achieve the 2D lattice, we propose that for this unique lattice symmetry there is a large energy barrier to thermodynamic out-of-plane growth, and a correspondingly large kinetic enhancement for lateral growth. These preferences for 2D vs. 3D growth of the hexagonal phase exist even at low CTAC concentrations and are independent of whether a substrate is present or not^50–53^.

Because entropic contributions to growth pathways are not considered in our model, we chose to experimentally test the prediction that 2D hexagonal chiral superlattices are kinetic products and their 3D analogues are thermodynamic products by assembling Td particles over considerably longer time periods, i.e. several days. Indeed, we observed the presence of large 3D structures consisting of alternating stacks of left and right planar chiral sheets and little-to-no 2D structures (Fig. 2h–i, Supplementary Fig. 20). Interestingly, images show the formation of nascent 2D island nuclei, consisting of clusters of 1, 2, or 4 Td particles (Fig. 2e–g). Note that once an out-of-plane adjacent trimer has formed (Fig. 2g), the binding of the 4^th^ particle is considerably more favorable than either top or side configurations (Fig. 2b). As a result, it is expected that 3 particle clusters would be short-lived, the lack of observation of which is consistent with our results. The broad range of times over which assembly is allowed to take place while still observing 2D superlattices (1 h to 2 days) and the long times necessary to observe 3D growth (5 days) is further evidence of the kinetic enhancement to planar Td assembly.

An additional factor that favors the observed 2D chiral hexagonal phase over alternative 3D structures (e.g., icosahedra) is the presence of a flat substrate onto which the superlattices may nucleate. Experimentally, we find that while a variety of different substrate materials support the formation of chiral hexagonal superlattices (Si, Si_3_N_4_, carbon, mica), assembly under identical conditions in the absence of a substrate results in disordered aggregates (Supplementary Fig. 21). Furthermore, assembly on the backside of a silicon wafer shows that the chiral Td phase can form on surfaces whose normal vector is oriented perpendicular to the direction of gravity, indicating that superlattices do not form in solution first and then sediment, but nucleate on the substrate because of attractive forces (Supplementary Fig. 22). Indeed, calculations from our model indicate that at low CTAC concentrations, Td face-substrate interactions are overwhelmingly attractive (i.e., d^* ^≈ 0 nm) but become significantly weaker when depletion forces become important at intermediate CTAC concentrations (i.e., d^*^ ≈ 9 nm, Supplementary Fig. 18). We hypothesize that the regime in which Td are strongly bound face-down to the surface is responsible for pulling particles out of solution and locally concentrating them, allowing for nucleation of 2D superlattice phases (Supplementary Fig. 23). This likely occurs via a similar mechanism discussed above for superlattice growth (Fig. 2c–h) but on a substrate rather than on an existing 2D superlattice, i.e. after a critical Td density is reached such that adjacent trimer configurations appear on the surface (red particles Fig. 2g), tip-down insertion of a 4^th^ Td is favorable (red particles Fig. 2h) and thus 2D assembled phases can nucleate.

To test this, we experimentally roughened Si substrates, which is known to weaken depletion interactions by reducing the favorable excluded volume gained by depletants^47^. Indeed, we observe that when the surface roughness exceeds the depletant size of ~5 nm, chiral Td superlattices either do not form at all or exist as small, disordered domains (Supplementary Fig. 24). This is consistent with our model, which indicates that a weakened depletion interaction between Td and substrates results in a nucleation regime that is shorter in duration and has weaker particle-particle interactions (Supplementary Fig. 18). Therefore, substrates act to seed heterogeneous nucleation of 2D Td superlattices, after which kinetically-enhanced lateral growth (Fig. 2) further disfavors the formation of 3D assemblies.

### Comparison of chiral and achiral 2D superlattices

**Fig. 3:**
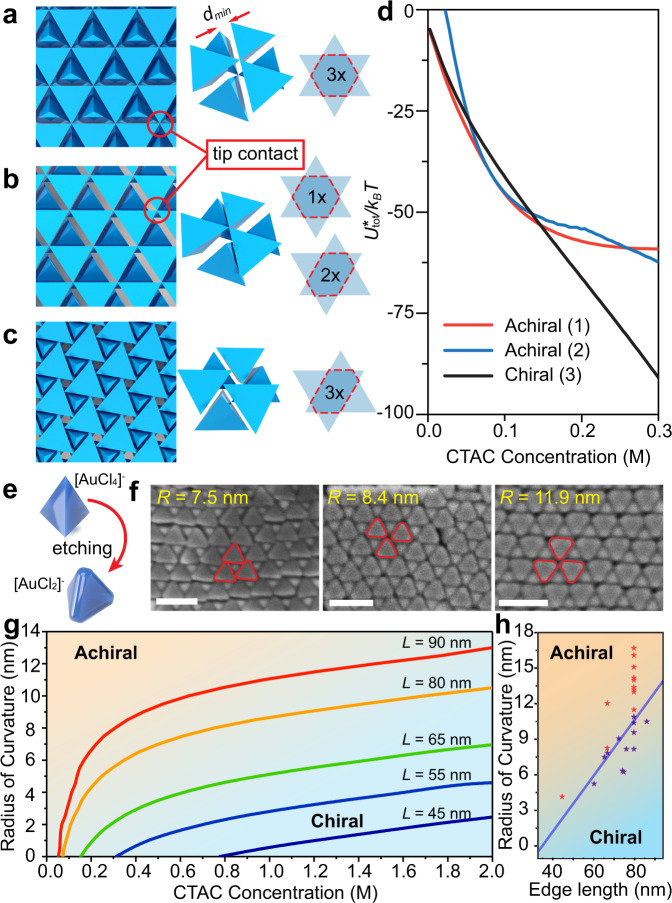
Understanding the stability of different 2D tetrahedra lattices. Schematic illustration of the lattice, hexagonal repeat unit, and particle-particle overlap area for: **a** achiral (1), **b** achiral (2), and **c** chiral (3) structures described in the text. The closest distance two particles can approach (*d*_*min*_) is set by the specific packing geometry. **d** Comparison of different superlattice energies as a function of hexadecyltrimethylammonium chloride (CTAC) concentration showing the ultimate stability of the chiral hexagonal (3) phase. **e** Illustration of the rounding of tips and edges as a result of selective oxidation of gold tetrahedra (Td) particles. **f** SEM images of the solvent evaporation-based assembly of Td particles with different tip radii (R), indicated in yellow. **g** Calculated concentration-dependent phase stability of achiral (1) and chiral (3) structures as a function of Td tip radius; separate lines are for Td with differing edge lengths. **h** Phase diagram predicting the stability of achiral (1) or chiral (3) structures at the endpoint of the assembly process based on particle size and tip radius; blue line is calculated phase boundary while red and purple dots indicate experimental observation of the achiral or chiral structures, respectively. All scale bars are 100 nm.

To understand the appearance of the chiral hexagonal structure, we next examined a series of related 2D superlattices of packed Td particles, all of which exhibit kinetically-enhanced lateral growth as discussed above (see SI, Supplementary Figs. 25 and 26). As a function of CTAC concentration, we compared the energy minimum of three different 2D phases: (1) an achiral hexagonal structure in which all three neighboring particles have identical hexagon-shaped overlap areas (Fig. 3a), (2) an achiral monoclinic structure with one nearest neighbor of hexagon-shaped overlap and two next-nearest neighbors with elongated hexagonal parallelogram-shaped overlap (Fig. 3b), (3) the chiral hexagonal structure discussed above with all three neighboring particles having identical elongated hexagonal parallelogram-shaped overlap (Fig. 3c). Of the three, the achiral structure (2) shows a preference for three-dimensional growth and is the least favorable at low-to-moderate CTAC concentrations and is therefore unlikely to be relevant to the formation of 2D chiral hexagonal phases (Fig. 3d and Supplementary Fig. 26). Interestingly, achiral structure (1) has the greatest facet overlap area and would therefore presumably pack into the lowest energy arrangement. However, as the equilibrium interparticle spacing (d^*^) decreases with increasing CTAC concentration, the tetrahedron tips eventually come into physical contact, sterically preventing further attraction between particles (Fig. 3a). Thus, a geometric constraint sets a minimum interparticle spacing (*d*_*min*_) that, if reached, severely limits further increases in the magnitude of the interaction potential (Fig. 3a, d and Supplementary Fig. 26). The consequence of this effect is that the chiral hexagonal phase (3) becomes more favorable than the achiral (1) phase at intermediate CTAC concentrations and remains the most stable structure until the solvent fully evaporates (Fig. 3d and Supplementary Fig. 27).

To test the importance of particle-based geometric constraints on the formation of the chiral hexagonal phase, we hypothesized that chemically truncating the tips of tetrahedra nanoparticles through a selective oxidation reaction would result in a decreased *d*_*min*_ and a more favorable achiral (1) phase^54–58^ (see SI section 2c, Fig. 3e and Supplementary Fig. 28). For tetrahedra with tips of a high radius of curvature (R), the achiral hexagonal structure (1) is indeed observed exclusively in electron microscopy images (Fig. 3f). We next corroborated this result by calculating the value of *d*_*min*_ for tetrahedra with tips of different radii of curvature and generated the resulting interaction potentials (Supplementary Fig. 29). The model indeed confirms that for particles with rounded tips, the decreased *d*_*min*_ and greater facet contact area allows for the achiral phase to become more favorable (Fig. 3g, Supplementary Figs. 29 and 30). Thus, for a particle of a given size, increasing the tip radius of curvature results in a transition from the chiral to the achiral phase (Supplementary Fig. 30). Although this phase transition point is dependent on the amount of CTAC present in solution (Fig. 3g), our electron microscopy images are collected when samples are completely dried and therefore represent a snapshot of the state of the system at an exceedingly high value of [CTAC] (rightmost part of Fig. 3g). We take this endpoint of the assembly process to be at ~2.0 M CTAC, as this is both the solubility limit of CTAC determined experimentally (Supplementary Fig. 31) and the point at which attractive interactions approach 10^4^ k_B_T when particle reorganization is exceedingly unlikely (Supplementary Fig. 14). Using this definition, the model allows us to generate a phase diagram that predicts whether achiral (1) or chiral (3) structures are preferred at the endpoint of the assembly process based on the morphological parameters of the particles (blue line Fig. 3h). Experimental electron microscopy images taken for Td particles of differing size and tip radius of curvature show excellent agreement with the model predictions (Fig. 3h, Supplementary Fig. 32). These findings indicate that the formation of chiral superlattices may be unusually sensitive to the geometry of the constituent building blocks^23^, highlighting the importance of synthesis and purification strategies that generate and maintain sharp-tipped particles (Fig. 1).

### Rotation of Td within planar chiral superlattices

**Fig. 4:**
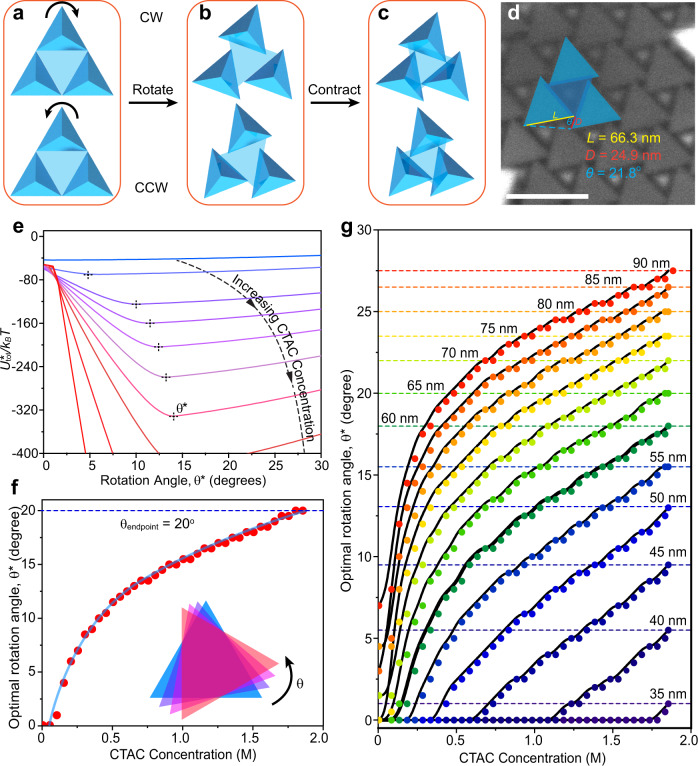
Particle rotation explains the achiral-chiral phase transition. **a** Tetrahedra (Td) packed into the achiral (1) structure may **b** undergo clockwise (CW) or counterclockwise (CCW) rotation to generate chiral enantiomers that **c** can form a denser lattice with more favorable interparticle attractions. **d** Definition of structural parameters for chiral superlattices including *L*, the Td edge length, *D*, the tip offset, and *θ*, the particle rotation angle. **e** Model calculations comparing the interaction potential (*U*_tot_^*^) as a function of rotation angle. Minimums in the plots define the optimal rotation angle (θ^*^), which shift to higher values with increasing hexdecyltrimethylammonium chloride (CTAC) concentration. **f** Optimal rotation angle for *L* = 66.3 nm edge length Td at different CTAC concentrations showing the predicted value at the endpoint of the assembly process of *θ*_*endpoint*_ = 20°. **g** Calculated optimal rotation angles (θ^*^) of chiral hexagonal assemblies for Td of varying edge length. Scale bar is 100 nm.

The comparison of different 2D tetrahedron superlattices indicates that although chiral structures are favorable at high CTAC concentrations (Supplementary Fig. 27), the achiral (1) structure is most stable at low CTAC concentrations (Fig. 3d). Since Td are assembled through slow evaporation and thus transition from low to high CTAC during crystallization, we hypothesized that, even for sharp-tipped particles, 2D superlattices first nucleate in the achiral (1) phase and then transition to the chiral (3) configuration (Supplementary Fig. 33). We propose a mechanism for this transition, in which Td packed into the achiral (1) structure may adopt chiral configurations via rotation about their [111] axis that is perpendicular to the plane of the 2D superlattice (Fig. 4a, b)^59^. This rotation would avoid the minimum spacing imposed by the achiral (1) phase (*d*_*min*_), allowing for closer interparticle distances and thus more favorable interactions (Fig. 4c). To evaluate the energetics of this process, we derived a geometric expression for *d*_*min*_ as a function of Td rotation angle, *θ* (see SI, Fig. 4d), allowing us to calculate equilibrium interaction energies (*U*_tot_^*^) for different values of *θ* over the range of CTAC concentrations (Fig. 4e); a rotation angle of 0° corresponds to the achiral (1) structure while all values between 0° and 30° indicate a morphology in the hexagonal chiral symmetry class. The consequence of Td rotation is a decreased *d*_*min*_, leading to closer surface-surface distances and more favorable interactions, but also a decreased facet contact area, leading to less favorable interactions. Thus, a balance between these influences results in an optimal rotation angle (θ^*^) at a particular CTAC concentration, given by the minimums in the curves shown in Fig. 4e. Plots of θ^*^ as a function of [CTAC] show the final rotation angle of Td chiral superlattices at the endpoint of the assembly process (i.e., ~2.0 M CTAC) which can be compared to experimental electron microscopy images; the measured value of ~21.8° shows excellent agreement with the prediction of ~20° for the 66.3 nm edge length Td particles used throughout most of this work (Fig. 4f). Since either clockwise or counterclockwise rotation will lower the energy of the system degenerately, there will emerge equal number of left and right enantiomers of 2D chiral crystals, consistent with our observations (Figs. 1d, 4a–c, Supplementary Fig. 15). Interestingly, extending this calculation to tetrahedra with a range of sizes shows that the emergence of the chiral phase via rotation is only favorable for Td with edge lengths above ~35 nm (Fig. 4g), which may explain why this symmetry has not been observed in previous reports on the assembly of tetrahedron-shaped semiconductor nanoparticles which tend to considerably smaller, on the order of 5–10 nm^11,34^.

## Discussion

These data allow for a stepwise formation mechanism to take shape for the assembly of 2D chiral Td superlattices as their interactions become move attractive over time during the evaporation of the solvent: (A) initial nucleation of the achiral (1) phase at low CTAC concentrations as a result of attractive particle-substrate interactions (Fig. 3), (B) kinetic enhancement of 2D lateral growth over out-of-plane growth, resulting in planar superlattices (Fig. 2), (C) densification of the achiral (1) phase until Td tips come into physical contact and set a minimum interparticle spacing (*d*_*min*_), sterically preventing the achiral (1) configuration from lowering its energy (Fig. 3), (D) nucleation of the chiral hexagonal phase within the achiral phase through random clockwise or counterclockwise rotation of Td, resulting in a closer interparticle spacing at the expense of a lower facet overlap area that nonetheless results in a net decrease in energy (Figs. 3 and 4), (E) further Td rotation according to the optimum value (θ^*^) until the final assembly endpoint is reached and samples are dried and static on substrates (Fig. 4).

These results establish the experimental parameters and fundamental mechanisms for the formation of 2D chiral superlattices. Whereas high-symmetry achiral building blocks, such as spheres and cubes, may assemble into chiral structures, they generally require the introduction of external fields or other chemical processes by which the symmetry of their interactions may be broken^2,12^. Alternatively, we have shown that tetrahedron-shaped particles, whose morphologies are intrinsically lower symmetry, can self-assemble into superlattices that exhibit spontaneous 2D chirality, contingent upon the presence of sharp tips after their synthesis and purification. Crystals with this geometry are absent from the extensive theoretical literature on the packing of tetrahedra, suggesting an even richer phase space than what was previously thought may remain to be discovered. Structures of this sort are particularly attractive as a means to generate chiroptical films and metamaterials with minimum human intervention at maximum production scale.

## Methods

### Chemical reagents

Hydrogen tetrachloroaurate hydrate (HAuCl_4_, trace metal basis), L-ascorbic acid (AA), sodium borohydride (NaBH_4_), silver nitrate (AgNO_3_, trace metal basis), hydrogen peroxide (H_2_O_2_, 30 wt%) and ammonia solution (NH_3_ ∙ H_2_O, 28–30 wt%) are all purchased from Sigma-Aldrich and hexadecyltrimethylammonium bromide (CTAB) and hexadecyltrimethylammonium chloride (CTAC) are purchased from TCI. All reagents are used as received. Milli-Q water (0.22 μM pore size, 18.2 MΩ ∙ cm at 25 °C) was used for all syntheses and purification and before each growth, all glassware were treated with aqua regia and rinsed with excess water.

### Synthesis of small gold seeds

The synthesis of small gold seeds follows Xia’s method^36^. First, HAuCl_4_•3H_2_O solution (10 mM, 250 μL) and 5 mL of 0.2 M CTAB solution are added to 4.75 mL Milli-Q water. Next 0.6 mL of freshly made ice-cold 10 mM NaBH_4_ solution was quickly injected into the above solution under vigorous stirring. The solution color changed from yellow to brownish-yellow, and the stirring was stopped after 2 min. This seed solution was then aged at 27 °C for 3 h before use.

### Synthesis of 10 nm seeds

Aqueous solutions of CTAC (200 mM, 20 mL), L- AA (100 mM, 15 mL), and an aqueous HAuCl_4_ solution (0.5 mM, 20 mL) were mixed, followed by injection of the initial, CTAB-capped Au seeds (500 μL)^36^. The reaction was allowed to continue at 27 °C for 15 min. The product was collected by centrifugation at 21300 rcf for 90 min, and then washed with water once and redispersed in 10 mM CTAC solution for further use and characterization. This 10 nm seed sample usually contains twinned products with the purity of single-crystalline seeds of ~85%, as shown in Supplementary Fig. 5.

### Synthesis of tetrahedra nanoparticles (Td NPs)

The synthesis of tetrahedra nanoparticles was adapted from Xia’s method with syringe pump to control the flow of an aqueous HAuCl_4_ solution^36^. In a typical synthesis of tetrahedra with edge length around ~66 nm, aqueous solutions of CTAC (200 mM, 27 mL), CTAB (200 mM, 9 mL), L-AA (100 mM, 36 mL), and the 10 nm seed solution (2.0 OD, 2 mL) were mixed with Milli-Q water (34 mL) in a 250 mL glass flask. Afterwards, aqueous HAuCl_4_ solution (0.5 mM, 60 mL) was drop-wisely injected by using a syringe pump at a rate of 6.0 mL/h. After the injection was completed the reaction was maintained at 27 °C for 10 min. Finally, the obtained Td NPs were collected by centrifugation at 12500 rcf for 15 min and then washed with water once to remove remaining reagents. The size of the tetrahedra can be controlled by the amounts of seeds in the growth solution and the volume of added HAuCl_4_ solution.

### Overgrowth of Au particles with Ag shells

After centrifugation, the products of the Au Td syntheses were then resuspended in CTAC (80 mM, 31.25 mL) in a 50 mL Falcon centrifuge tube. Afterwards, L-AA (100 mM, 5.25 mL) and AgNO_3_ (10 mM, 12.5 mL) were added and mixed thoroughly. This mixture was heated at 65 °C for 4 h with a shaking rate of 1000 rpm. After overgrowth, the Au Td NPs were embedded in Ag cubes, decahedra NPs formed Ag nanorods (NRs), and bitetrahedra NPs became Ag right bipyramid (RBP) NPs (Supplementary Fig. 7). The Au decahedra@Ag nanorods precipitate from the growth solution allowing for the remaining supernatant to be collected.

### Purification of Au Td@Ag cube NPs

To improve the purity of Au Td@Ag cube NPs, particle self-assembly was used to selectively precipitate impurities. For the synthesized Au Td@Ag cubes mentioned above, the edge length is around 70 nm. Based on an empirical interaction potential equation (see SI for calculations), when the energy reaches 4–5 *k*_*B*_*T*, NPs with large contact area will assemble into extended structures and precipitate from solution. Based on these calculations, the impure Au Td@Ag cube NP samples were suspended in CTAC (35 mM, 40 mL) at room temperature. Assemblies precipitated out overnight and the supernatant containing impurities was carefully removed. The selectively assembled Au Td@Ag cube NPs were then re-dispersed in water and brough to a CTAC concentration of 32 mM to allow for further rounds of purification. Four cycles of selective precipitation were necessary to reach >95% purity of Au Td@Ag cube NPs.

### Selective etching of Ag shells on Au Td@Ag cube NPs

To remove the Ag shell on purified Au Td@Ag cube NPs, samples were re-dispersed in CTAB (50 mM, 20 mL), followed by addition of NH_3_ ∙ H_2_O solution (28–30 wt%, 1 mL) and H_2_O_2_ (30 wt%, 1 mL). The oxidation reaction driven by NH_3_ ∙ H_2_O/H_2_O_2_ has been widely used in selective etching of Ag and showed no impact on the shape of the final Au Td particles. Indeed, it is likely that the formation of a AuAg surface alloy after the overgrowth creates a tolerance for the NH_3_ ∙ H_2_O/H_2_O_2_ etching solution, facilitating preservation and stabilization of the sharp tips of Au Td NPs. After etching at room temperature overnight, the sample was centrifuged at 13500 rcf for 15 min and then washed with water once to remove excess ammonia/H_2_O_2_ solution. The Au Td NP pellet was resuspended in 50 mM CTAC and aged at room temperature for 12 h to ensure exchange of CTAB to CTAC. This solution was centrifuged at 13500 rcf for 15 min and washed with 10 mM CTAC twice for further use.

### Controlled rounding of the tips of Au Td NPs

Purified Au Td NPs were suspended in 50 mM CTAB at a particle concentration of 2 OD at *λ*_max_ (~643 nm). To control the radius of curvature, different volumes of HAuCl_4_ solution (1 mM) were added to 0.5 mL Au Td NP samples in 1.5 mL centrifuge tubes. The final concentration of Au^3+^ was 1 μM, 2 μM, and 3 μM which drives a mild but tip-selective comproportionation reaction^54–58^. After etching at 40 °C for 4 h, rounded Au Td NPs with tips of different radii of curvature were obtained. Samples were centrifuged at 13500 rcf for 3 min to remove excess reagent, aged in 50 mM CTAC overnight to ensure CTAB to CTAB ligand exchange, and then washed with 10 mM CTAC twice for further use.

### Assembly of Au Td NPs on TEM grids

If purified Au Td NPs are allowed to dry slowly, excess CTAC will crystalize on the TEM grid, decreasing the image quality. To avoid this problem, Au Td NPs in CTAC solution (~2 OD, 100 μL) with water (1.4 mL) were centrifuged once to remove the excess CTAC and the obtained pellet was re-suspended in diluted CTAC solution (5 mM, 20 μL). A ~10 μL drop of this solution was placed on the TEM grid and allowed to evaporate at room temperature. Since the concentration of remaining CTAC increases during evaporation, the high values necessary to favor the chiral hexagonal phase of Td NPs are still reached. Before taking images, the TEM grid was rinsed carefully with chloroform to remove excess CTAC and increase the contrast of corresponding TEM images.

### Assembly of Au Td NPs on silicon wafers

Silicon wafers were first washed with acetone, isopropanol, and water with ultrasonication for 10 min each and dried with nitrogen gas. The clean silicon wafers were transferred into a small petri dish (diameter of 5 cm) and purified Au Td NPs in 5 mM CTAC (15 μL, 10 OD) were carefully pipetted onto the surface of a silicon wafer. To decrease the evaporation rate and increase the quality of the assemblies, samples at room temperature (21 ºC) were placed inside of a glass petri dish (15 cm in diameter) with two petri dishes (5 cm in diameter) of water (5 mL for each) to increase the humidity (Supplementary Fig. 13). Once dry, crystalized CTAC was removed by adding 10 μL of chloroform onto the silicon wafer and wicking it away with filter paper. After 2–3 times, a golden film is observed indicating exposure of the Au Td NP assemblies.

### Roughening of silicon wafers

Clean silicon wafers were roughened by reactive ion etching (RIE, power of 100 W) under CF_4_/oxygen (10/5 sccm) gases with a pressure of 250 mTorr using different etching times (from 1 to 5 min). Surface roughness was measured by The DektakXT® stylus profilometer.

### Interaction potential model

The theoretical model developed for tetrahedron assembly considered the net interaction potential between particles to be the additive contribution from van der Waals (*vdW*), electrostatic (*el*), and depletion forces (*dep*). To simplify the geometry of the model, we approximate the total potential for a single particle to be the sum of individual facet interaction energies:

1 $${U}_{{tot}}=\mathop{\sum}\limits_{{Facets}}{U}^{{Face}-{Face}}=\mathop{\sum}\limits_{{Facets}}\left({U}_{{vdW}}^{{Face}-{Face}}+{U}_{{el}}^{{Face}-{Face}}+{U}_{{dep}}^{{Face}-{Face}}\right)$$

Because the tetrahedra used in this work are bounded by flat, crystallographically-well-defined surfaces, this allows for simplified expressions for the van der Waals, electrostatic, and depletion forces that consider the interaction of two planar surfaces each with an area, *A*. This method of decomposing the energy of a polyhedral particle into its individual flat facet interactions is appropriate under the present conditions because all of the forces involved are short-ranged relative to the ~70 nm particle size. Thus, considering only nearest-neighbor effects is sufficient and the interaction energy per particle is proportional to the crystal energy. For example, the high electrolyte concentration (2.0 M) creates an inverse Debye screening length <1 nm, the micelle diameter (which sets the maximum distance of the depletion interaction) is ~5 nm (Supplementary Fig. 16b), and 90% of the van der Waals attraction can be captured by only considering the first 5 nm of depth of the Au nanoparticle (Supplementary Fig. 17). Therefore, the fundamental length scales defining each component of the potential imply that all can be predominantly relegated to the surface of the particle, allowing for the overlap area, *A*, to become the focus of the calculations. Finally, because the particles used in this work have been purified in such a way as to prevent rounding of their tips and edges, the majority of the tetrahedron surface area consists of flat {111} facets. Under this model, all interparticle forces are linearly proportional to *A*, which is appealing because it simplifies the task of comparing the energies of different particle configurations to a geometric calculation for the overlap area of neighboring particle faces (Supplementary Fig. 1-4).

We interpret the nearest-neighbor single-particle interaction potential (*U*_*tot*_) as being proportional to the thermodynamic free energy of a Td superlattice following a set of simple assumptions that are foundational to the calculation of crystal properties in statistical mechanics^46^. First, when comparing two or more different crystal structures, entropic effects are ignored. This is valid because the entropy of crystallization is dominated by the reduced translational or rotational degrees of freedom when particles transition from a gas or liquid state to a solid; any entropic differences due to the specific crystalline structure (e.g., achiral or chiral hexagonal) are negligible by comparison. Second, the Helmholtz free energy (*F*):

2 $${F}_{{crystal}}={U}_{{crystal}}-T{S}_{{crystal}}$$

shows that at constant temperature (*T*), if the entropy (*S*_*crystal*_) is ignored, the overall interaction energy of the crystal (*U*_*crystal*_) is proportional to the free energy (*F*_*crystal*_). Finally, if the individual elements in the crystal (atoms or nanoparticles) experience interactions that are short-ranged relative to their size, the nearest-neighbor environment is all that is needed to capture the energy of the lattice (i.e., next nearest neighboring interactions can be ignored). As discussed above, the length scale of Td particle interactions extend at most to ~10% of the particle size and can therefore be considered to be sufficiently short-ranged. The consequence of this line of reasoning is that comparing the relative *U*_*tot*_ value for single Td particles in different superlattice configurations is equivalent to comparing the relative thermodynamic free energy of their crystals.

van der Waals interactions are given by^38^:

3 $${U}_{{vdW}}^{{Face}-{Face}}=\frac{-{AH}}{12\pi {d}^{2}}$$

where *d* is the separation between flat particle facets, *A* is the particle–particle facet overlap area, and *H* is the Hamaker constant, taken to be 40 × 10^−20 ^*J* for a Au-water-Au geometry according to ref. ^60^.

Electrostatic interactions are given by the solution of the linearized Poisson–Boltzmann equation, assuming no charge regulation^61^:

4 $${U}_{{el}}^{{Face}-{Face}}=\varepsilon {\varepsilon }_{0}\kappa {\varphi }^{2}A\left[1-{{{{{\rm{tanh }}}}}}\left(\kappa \frac{d-2{t}_{{CTAC}}}{2}\right)\right]$$

where *ε* is the relative permittivity of the solvent (water) and *ε*_*0*_ the permittivity of free space, $\varphi$ is the constant potential at the CTAC-modified nanoparticle surface taken to be 0.035 V according to ref. ^62^, *t*_*CTAC*_ the thickness of the CTAC bilayer taken to be 3.2 nm according to refs. ^39,63^, and *κ* the inverse Debye length which for a monovalent electrolyte is given by^38^:

5 $$\kappa ={\left(\frac{c{e}^{2}}{\varepsilon {\varepsilon }_{0}{k}_{B}T}\right)}^{1/2}$$

where *c* is the salt concentration, *e* the elementary charge, *k*_*B*_ Boltzmann’s constant, and *T* temperature. To calculate the ionic strength *c*, we consider CTAC to contribute with unity up to the critical micelle concentration (CMC = 1.31 mM for CTAC)^64^, after which the fractional charge of micelles (*α* = 0.28 for CTAC)^65^ is used to account for incomplete micelle counterion dissociation, which is approximately concentration independent^65^.

Depletion interactions are given by^40^:

6 $${U}_{{dep}}^{{Face}-{Face}}=-\Delta \Pi A(2{t}_{{CTAC},{eff}}+{D}_{{CTAC},{eff}}-d)$$

where *t*_*CTAC,eff*_ and *D*_*CTAC,eff*_ represent an effective size for the CTAC bilayer thickness and micelle diameter, respectively, which is larger than their physical size because they are electrostatically charged. This is accounted for by adding a factor *δ* multiplied by the Debye length κ^−1^ according to ref. ^41^:

7 $${t}_{{CTAC},{eff}}={t}_{{CTAC}}+\,\delta {\kappa }^{-1}$$

8 $${D}_{{CTAC},{eff}}={D}_{{CTAC}}+\,2\delta {\kappa }^{-1}$$

where *t*_*CTAC*_ and *D*_*CTAC*_ are the physical sizes of the CTAC bilayer thickness and micelle diameter, respectively. We take *δ* = 0.725, determined for a similar system of quaternary ammonium halide coated gold nanoparticles assembled via charged quaternary ammonium surfactant depletants according to ref. ^66^. *D*_*CTAC*_ is calculated according to the Israelachvilli molecular packing parameter model:

9 $${{D}_{{CTAC}}=2\left(\frac{3{N}_{{agg}}{V}_{0}}{4\pi }\right)}^{1/3}$$

where *V*_*0*_ is the molecular volume equal to 0.4309 nm^3^ for CTAC according to ref. ^67^, and *N*_*agg*_ is the aggregation number for CTAC micelles. We account for the possibility of *D*_*CTAC*_ changing with CTAC concentration by fitting the concentration-dependent *N*_*agg*_ values from ref. ^65^ to an empirical power-law function with *R*^2^ = 0.986.

ΔΠ is the change in osmotic pressure given by the Carnahan-Starling equation of state for a system of concentrated charged micelles by^61,68^:

10 $$\Delta \Pi =n{k}_{B}T\left(1+{\phi }_{{eff}}+{\phi }_{{eff}}^{2}-{\phi }_{{eff}}^{3}\right){(1-{\phi }_{{eff}})}^{-3}$$

where *n* = (*N*_*A*_/*N*_*agg*_)(*c*-CMC) and represents the micelle concentration, *N*_*A*_ is Avogadro’s number and

11 $${\phi }_{{eff}}=n\frac{4}{3}\pi {\left(\frac{{d}_{{CTAC},{eff}}}{2}\right)}^{3}$$

following the approach of ref. ^41^.

Using the above framework, the evolution of a system of CTAC-coated particles from monomeric entities to assembled superlattices can be predicted by: (1) calculating the total energy per particle, ${U}_{{tot}}$, as a function of interparticle separation, *d*, (2) finding the minimum (equilibrium) value of the potential, ${U}_{{tot}}^{* }$, and corresponding equilibrium spacing d^*^, and (3) iterating this process as the concentration of depletants (CTAC micelles) and electrolytes (CTA^+^ and Cl^−^ ions) increases as a result of solvent evaporation. Since increasing depletants makes particles more attractive and increasing electrolytes make particles less repulsive, the magnitude of the interaction gradually increases (i.e., more negative ${U}_{{tot}}^{* }$) and the equilibrium spacing (d*) gradually decreases as the sample droplet shrinks over time. Comparing several such calculations for tetrahedra assembled into different geometric configurations (with corresponding overlap areas, *A*) allows for determination of the lowest energy superlattice. We consider the assembly process complete when a CTAC concentration of ~2.0 M is reached, as this is approximately the solubility limit for CTAC in water (Supplementary Fig. 31) and the point at which the sample has less than 1% of the initial volume. This results in a system with interaction potentials that are so strongly attractive (1000’s of k_B_T) that all particles are irreversibly attractive and the possibility for rearrangement is minimal (Supplementary Fig. 18).

The interaction between the substrate and the tetrahedra is treated separately from particle-particle interactions but modeled using same framework as above. For vdW interactions, a Hamaker constant for a Au-water-Si geometry was approximated to be 10 × 10^−20 ^*J* using the combining relationships and data from several references ^61,69,70^. For electrostatic interactions, a similar linearized Poisson–Boltzmann equation was used but for two dissimilar surfaces^61^:

12 $${U}_{{el}}^{{Face}-{Sub}}=\varepsilon {\varepsilon }_{0}\kappa {\varphi }_{{Au}}{\varphi }_{{Si}}A\left[1-{{{{{\rm{tanh }}}}}}\left(\kappa \frac{d-2{t}_{{CTAC}}}{2}\right)\right]$$

where ${\varphi }_{{Au}}$ is the constant potential at the CTAC-modified gold surface (defined above) and ${\varphi }_{{Si}}$ is the constant potential at the CTAC-modified silicon substrate, taken to be 0.004 V according to ref. ^70^. Depletion forces are assumed to remain unchanged for NP-substrate interactions for which the Si surface has a roughness less than the depletant micelle size of ~5 nm; this is the case for all experimental results unless otherwise noted. When roughness exceeds the depletant size, depletion forces are weakened because asperities prevent the closest approach of surfaces and thereby reduce the excluded volume that drives the attraction^47^. We develop a qualitative understanding of the importance of this effect by assuming that depletion forces are reduced to 1/10 their original magnitude in the presence of roughened silicon substrates, which is consistent with literature results (see Supplementary Figs. 23 and 24 for more details)^47^.

Because the 2D superlattices reported in this work are observed to form on a range of different flat and homogeneous substrate materials (e.g., Si, Si_3_N_4_, carbon, mica, Supplementary Fig. 21), regardless of whether they are plasma cleaned or not, we conclude that wetting and/or contact angle pinning effects are not important contributors to the assembly mechanism.

### Characterization

Transmission electron microscopy (TEM) was performed with a JOEL 2100 F at a voltage of 200 kV in TEM mode. The high angle angular dark field-scanning transmission electron microscope (HAADF-STEM) images were collected by the FEI Titan Themis^3^ S/TEM operated at an accelerating voltage of 300 kV with a double tilt holder. Scanning electron microscope (SEM) images were collected with a FEI Helios NanoLab 660 Dual Beam with working voltage of 5 kV, working current of 25 pA, and working distance of 4 mm.

### Image analysis and structure assignment

Shape purity analysis of Au Td NPs was performed using Adobe Photoshop by marking tetrahedron-shaped NPs with red dots and impurities with blue dots and exporting their count values. Edge lengths were measured by the Gatan microscopy suite (GMS 3.0) software with the original.dm3 files. The radius of curvature of truncated Td NPs was analyzed with Adobe Illustrator using the rounded triangular shape tool which, when calibrated to the image scale bar, allowed for calculation of a quantitative value. All 3D models of Td superlattices were constructed and rendered with Cinema 4D.

## Supplementary information

Supplementary Information

## Data Availability

The electron microscopy and interaction potential model datasets generated during and/or analyzed during the current study are available in the Supplementary Information file. Additional images and raw model data are available from the corresponding author on request.

## Code availability

The MATLAB code used to calculate all interparticle potentials is available from the corresponding author on request.

## References

[1] Cecconello A, Besteiro LV, Govorov AO, Willner I (2017). Chiroplasmonic DNA-based nanostructures. Nat. Rev. Mater..

[2] Mastroianni AJ, Claridge SA, Alivisatos AP (2009). Pyramidal and chiral groupings of gold nanocrystals assembled using DNA scaffolds. J. Am. Chem. Soc..

[3] Yashima E (2016). Supramolecular helical systems: helical assemblies of small molecules, foldamers, and polymers with chiral amplification and their functions. Chem. Rev..

[4] Hentschel M, Schäferling M, Duan X, Giessen H, Liu N (2017). Chiral plasmonics. Sci. Adv..

[5] Kuzyk A (2014). Reconfigurable 3D plasmonic metamolecules. Nat. Mater..

[6] Zhou C, Duan X, Liu N (2015). A plasmonic nanorod that walks on DNA origami. Nat. Commun..

[7] Mammana A, D’Urso A, Lauceri R, Purrello R (2007). Switching off and on the supramolecular chiral memory in porphyrin assemblies. J. Am. Chem. Soc..

[8] Nagaoka Y (2018). Superstructures generated from truncated tetrahedral quantum dots. Nature.

[9] Zerrouki D, Baudry J, Pine D, Chaikin P, Bibette J (2008). Chiral colloidal clusters. Nature.

[10] Chen Q (2011). Supracolloidal reaction kinetics of Janus spheres. Science.

[11] Srivastave S (2010). Light-controlled self-assembly of semiconductor nanoparticles into twisted ribbons. Science.

[12] Singh G (2014). Self-assembly of magnetite nanocubes into helical superstructures. Science.

[13] Pendry JB (2004). A chiral route to negative refraction. Science.

[14] Soukoulls CM, Wegener M (2011). Past achievements and future challenges in the development of three-dimensional photonic metamaterials. Nat. Photonics.

[15] Agarwal A, Lilly GD, Govorov AO, Kotov NA (2008). Optical emission and energy transfer in nanoparticle−nanorod assemblies: potential energy pump system for negative refractive index materials. J. Phys. Chem. C..

[16] Smith KW (2018). Exploiting evanescent field polarization for giant chiroptical modulation from achiral gold half-rings. ACS Nano.

[17] Hendry E (2010). Ultrasensitive detection and characterization of biomolecules using superchiral fields. Nat. Nanotech..

[18] Papakostas A (2003). Optical manifestations of planar chirality. Phys. Rev. Lett..

[19] Li Z, Gokkavas M, Ozbay E (2013). Manipulation of asymmetric transmission in planar chiral nanostructures by anisotropic loss. Adv. Opt. Mater..

[20] Schnell M (2016). Real-space mapping of the chiral near-field distributions in spiral antennas and planar metasurfaces. Nano Lett..

[21] Ogier R, Fang Y, Svedendahl M, Johansson P, Käll M (2014). Macroscopic layers of chiral plasmonic nanoparticle oligomers from colloidal lithography. ACS Photonics.

[22] Damasceno PF, Engel M, Glotzer SC (2012). Crystalline assemblies and densest packings of a family of truncated tetrahedra and the role of directional entropic forces. ACS Nano.

[23] Van Damme R, Coli GM, Van Roij R, Dijkstra M (2020). Classifying crystals of rounded tetrahedra and determining their order parameters using dimensionality reduction. ACS Nano.

[24] Chen ER, Engel M, Glotzer SC (2010). Dense crystalline dimer packings of regular tetrahedra. Discret. Comput. Geom..

[25] Haji-Akbari A (2009). Disordered, quasicrystalline and crystalline phases of densely packed tetrahedra. Nature.

[26] Conway JH, Torquato S (2006). Packing, tiling, and covering with tetrahedra. Proc. Natl Acad. Sci. USA.

[27] Lee H-E (2018). Amino-acid-and peptide-directed synthesis of chiral plasmonic gold nanoparticles. Nature.

[28] Im SW (2020). Chiral surface and geometry of metal nanocrystals. Adv. Mater..

[29] Kim H (2020). γ‐Glutamylcysteine‐and cysteinylglycine‐directed growth of chiral gold nanoparticles and their crystallographic analysis. Angew. Chem. Int. Ed..

[30] Jiang W (2020). Emergence of complexity in hierarchically organized chiral particles. Science.

[31] Walker DA, Leitsch EK, Nap RJ, Szleifer I, Grzybowski BA (2013). Geometric curvature controls the chemical patchiness and self-assembly of nanoparticles. Nat. Nanotech..

[32] Smith KW (2016). Chiral and achiral nanodumbbell dimers: the effect of geometry on plasmonic properties. ACS Nano.

[33] Nagaoka Y, Zhu H, Eggert D, Chen O (2018). Single-component quasicrystalline nanocrystal superlattices through flexible polygon tiling rule. Science.

[34] Boles MA, Talapin DV (2014). Self-assembly of tetrahedral CdSe nanocrystals: Effective “patchiness” via anisotropic steric interaction. J. Am. Chem. Soc..

[35] Sun M, Cheng Z, Chen W, Jones MR (2021). Understanding symmetry breaking at the single-particle level via the growth of tetrahedron-shaped nanocrystals from higher-symmetry precursors. ACS Nano.

[36] Zheng Y (2014). Seed‐mediated synthesis of gold tetrahedra in high purity and with tunable, well‐controlled sizes. Chem. Asian J..

[37] Kim F, Connor S, Song H, Kuykendall T, Yang P (2004). Platonic gold nanocrystals. Angew. Chem. Int. Ed..

[38] Israelachvili, J. N. Intermolecular and surface forces, 3rd edition. (Academic Press, Cambridge, MA, 2011).

[39] Pashley RM, McGuiggan PM, Horn RG, Ninham BW (1988). Forces between bilayers of cetyltrimethylammonium bromide in micellar solutions. J. Colloid Interface Sci..

[40] Bishop KJM, Wilmer CE, Soh S, Grzybowski BA (2009). Nanoscale forces and their uses in self-assembly. Small.

[41] Iracki TD, Beltran-Villegas DJ, Eichmann SL, Bevan MA (2010). Charged micelle depletion attraction and interfacial colloidal phase behavior. Langmuir.

[42] Hueckel T, Hocky GM, Palacci J, Sacanna S (2020). Ionic solids from common colloids. Nature.

[43] Henzie J, Grünwald M, Widmer-Cooper A, Geissler PL, Yang P (2012). Self-assembly of uniform polyhedral silver nanocrystals into densest packings and exotic superlattices. Nat. Mater..

[44] Coropceanu I (2022). Self-assembly of nanocrystals into strongly electronically coupled all-inorganic supercrystals. Science.

[45] Vutukuri HR, Badaire S, Matthijs de Winter DA, Imhof A, van Blaaderen A (2015). Directed self-assembly of micron-sized gold nanoplatelets into oriented flexible stacks with tunable interplate distance. Nano Lett..

[46] McQuarrie, D. A. Statistical mechanics. (Univ. Sci. Books, California, 2015).

[47] Zhao K, Mason TG (2007). Directing colloidal self-assembly through roughness-controlled depletion attractions. Phys. Rev. Lett..

[48] Young KL (2012). Assembly of reconfigurable one-dimensional colloidal superlattices due to a synergy of fundamental nanoscale forces. Proc. Natl Acad. Sci. USA.

[49] van Anders G, Klotsa D, Ahmed NK, Engel M, Glotzer SC (2014). Understanding shape entropy through local dense packing. Proc. Natl Acad. Sci. USA.

[50] Nakagawa Y, Kageyama H, Oaki Y, Imai H (2014). Direction control of oriented self-assembly for 1D, 2D, and 3D microarrays of anisotropic rectangular nanoblocks. J. Am. Chem. Soc..

[51] Fendler JH (2001). Chemical self-assembly for electronic applications. Chem. Mater..

[52] Dong A, Chen J, Vora PM, Kikkawa JM, Murray CB (2010). Binary nanocrystal superlattice membranes self-assembled at the liquid–air interface. Nature.

[53] Ye X (2013). Competition of shape and interaction patchiness for self-assembling nanoplates. Nat. Chem..

[54] O’Brien MN, Jones MR, Kohlstedt KL, Schatz GC, Mirkin CA (2015). Uniform circular disks with synthetically tailorable diameters: two-dimensional nanoparticles for plasmonics. Nano Lett..

[55] Jones MR (2017). Deterministic symmetry breaking of plasmonic nanostructures enabled by DNA-programmable assembly. Nano Lett..

[56] O’Brien MN, Jones MR, Brown KA, Mirkin CA (2014). Universal noble metal nanoparticle seeds realized through iterative reductive growth and oxidative dissolution reactions. J. Am. Chem. Soc..

[57] Bhattarai A (2020). Tip-enhanced Raman nanospectroscopy of smooth spherical gold nanoparticles. Phys. Chem. Lett..

[58] Wang C-F (2020). Tip-enhanced multipolar Raman scattering. Phys. Chem. Lett..

[59] Gantapara AP, Qi W, Dijkstra M (2015). A novel chiral phase of achiral hard triangles and an entropy-driven demixing of enantiomers. Soft Matter.

[60] Leite FL, Bueno CC, Da Róz AL, Ziemath EC, Oliveira ON (2012). Theoretical models for surface forces and adhesion and their measurement using atomic force microscopy. Int. J. Mol. Sci..

[61] Hunter, R. J. Foundation of colloid science (Oxford Univ. Press, New York, 594–598, 2001).

[62] Walker DA, Browne KP, Kowalczyk B, Grzybowski BA (2010). Self-assembly of nanotriangle superlattices facilitated by repulsive electrostatic interactions. Angew. Chem. Int Ed..

[63] Alkilany AM, Frey RL, Ferry JL, Murphy CJ (2008). Gold nanorods as nanoadmicelles: 1-Naphthol partitioning into a nanorod-bound surfactant bilayer. Langmuir.

[64] Asakawa T, Kitano H, Ohta A, Miyagishi S (2001). Convenient estimation for counterion dissociation of cationic micelles using chloride-sensitive fluorescence probe. J. Colloid Interface Sci..

[65] Aswal VK, Goyal PS (2003). Role of different counterions and size of micelle in concentration dependence micellar structure of ionic surfactants. Chem. Phys. Lett..

[66] Young KL (2012). Assembly of reconfigurable one-dimensional colloidal superlattices due to a synergy of fundamental nanoscale forces. Proc. Natl Acad. Sci. USA.

[67] Shah SK, Bhattarai A (2020). Interfacial and micellization behavior of cetyltrimethylammonium bromide (CTAB) in water and methanol-water mixture at 298.15 to 323.15 K. J. Chem..

[68] Carnahan NF, Starling KE (1969). Equation of state for nonattracting rigid spheres. J. Chem. Phys..

[69] Rehn SM (2021). Mechanical reshaping of inorganic nanostructures with weak nanoscale forces. Nano Lett..

[70] Rouhollahi A, Fazlolahzadeh O, Dolati A, Ghahramanifard F (2018). Effects of different surfactants on the silica content and characterization of Ni–SiO_2_ nanocomposites. J. Nanostruct. Chem..

