# Supplementary material for: Assembly of planar chiral superlattices from achiral building blocks
Source: Nat Commun. 2022 Jul 21;13:4207. doi: 10.1038/s41467-022-31868-2 (PMC9304327; doi:10.1038/s41467-022-31868-2)
Supplement: Supplementary file 1 — Supplementary Information [file 41467_2022_31868_MOESM1_ESM.pdf]

Supplementary Information

Assembly of Planar Chiral Superlattices from Achiral Building Blocks

Zhihua Cheng,[1] Matthew R. Jones[1,2*]

Department of Chemistry,[1] Department of Materials Science & Nanoengineering,[2] Rice University, Houston, TX, 77005, United States

Table of contents

Supplementary calculations and discussion

1. Assembly of Au Td@Ag cube NPs for purification
2. Tetrahedron structure analysis and calculations
   2.a. For achiral (1) assemblies
   2.b. For achiral (2) assemblies
   2.c. For the packing of tetrahedra with rounded tips
   2.d. For the transition from achiral (1) to chiral (3) via rotation-based phase change

Supplementary figures

Supplementary Calculations and Discussion

<u>1. Assembly of Au Td@Ag cube NPs for purification</u>: Self-assembly has been widely used to purify NPs with large surface area since they can form extended assemblies and precipitate (e.g., cubes, prisms, high aspect ratio nanorods, etc)[1-3]. Since the target Au Td NPs could be selectively embedded in Ag cubes *via* overgrowth, we hypothesized they could be purified by considering the assembly of cube-shaped particles. To determine the purification conditions, especially the concentration of surfactant, we used the following empirical formula for interparticle attraction that has been used in previous reports to predict selective assembly and precipitation[1-3]:

$$\frac{|U|}{k_B T} = \frac{2 r_m A N_0 (c - cmc)}{n} \quad \text{(Supplementary eq. 1)}$$

where k_BT is the thermal energy, r_m the micelle radius, *A* the particle-particle facet contact area, N_0 Avogadro's number, *c* the concentration of surfactant, *cmc* the critical micelle concentration, and *n* the number of molecules that assemble to form a micelle. For CTAC solutions, the r_m, *cmc* and *n* are 3 nm, 1 mM and 120, respectively. For a typical Au Td@Ag cube NP, the cube edge length is 70 nm, leading to a possible contact area, A, of 4900 nm^2. According to previous reports, when the empirical potential |U| reaches 4~5k_BT, the targeted NPs will assemble and precipitate, suggesting that a CTAC concentration around 28 - 35 mM will result in Au Td@Ag cube NP assembly. It is important to mention that due to the batch-to-batch differences, it is necessary to measure the size of Au Td@Ag cube NPs before purification and adjust conditions accordingly; most of our samples required 32 mM CTAC to purify Au Td@Ag cube NPs with 70 nm edge length. Multiple rounds of precipitation and separation with this method lead to increasingly pure samples.

<u>2. Tetrahedron structure analysis and calculations</u>: To have a complete understanding of the formation of the chiral hexagonal (3) phase, we considered a series of related 2D structures with different packing of Td particles. Due to the nature of the interactions between particles in our system, assemblies that have the highest face-to-face contact area and smallest interparticle distance will be those of minimum energy, i.e., most favorable. Since we are only considering structures capable of forming extended 2D crystals, we exclude from our analysis Td assemblies that result in local decahedral or icosahedral packing and lack long-range order.

<u>2.a. For achiral (1) assemblies</u>: we consider a structure where a tetrahedron dimer with hexagon-shaped face-to-face contact area is arrayed in a 2D hexagonal Bravais lattice (Supplementary Figure 1). The extended material resembles two interpenetrated sheets of Td, each with in-plane triangular tip-to-tip packing (Supplementary Figure 1A, 1B). This results in a hexagonal contact area (Supplementary Figure 1C, 1D) and interparticle distance, *d* (Supplementary Figure 1E).

For tetrahedra with edge length (*L*), the overlap area (*S*) in Supplementary Figure 1D is given by a hexagon with edge length of L/3:

$$S = \frac{\sqrt{3}}{6} L^2 \quad \text{(Supplementary eq. 2)}$$

The minimum interparticle distance (d_{min}) can be obtained with the model in Supplementary Figure 1B using the equations of two planes. We consider a cube with edge length *a*, the diagonal of which produces a Td with edge length *L*. Two typical planes as shown in Supplementary Figure 1C and 1E can be calculated by two sets of points:

Plane I: $(a, a, 0); (a, 0, a); (0, a, a)$

Plane II: $\left(\frac{a}{2}, \frac{a}{2}, \frac{3a}{2}\right); \left(\frac{3a}{2}, \frac{a}{2}, \frac{a}{2}\right); \left(\frac{a}{2}, \frac{3a}{2}, \frac{a}{2}\right)$

The corresponding equations for above planes are:

Plane I: $x + y + z = 2a$

Plane II: $x + y + z = 2.5a$

The distance (*d*) between two parallel planes is:

$$d = \frac{|2.5a - 2a|}{\sqrt{1^2 + 1^2 + 1^2}} = \frac{a}{2\sqrt{3}} = \frac{\sqrt{3}a}{6} \qquad \text{(Supplementary eq. 3)}$$

Since the edge length (*L*) of tetrahedra is:

$$L = \sqrt{2}a \qquad \text{(Supplementary eq. 4)}$$

and since this is the closest-packed Td can get in the achiral (1) phase given their tip sharpness, the minimum distance (d_{min}) between two tetrahedra NPs is:

$$d_{min} = d = \frac{\sqrt{3}}{6}a = \frac{\sqrt{3}}{6}\frac{\sqrt{2}}{2}L = \frac{\sqrt{6}}{12}L \qquad \text{(Supplementary eq. 5)}$$

For the tetrahedra in this work, L = 66.3 nm, so the corresponding S and d_{min} are:

S = 1268.926 nm^2

d_{min} = 13.533 nm

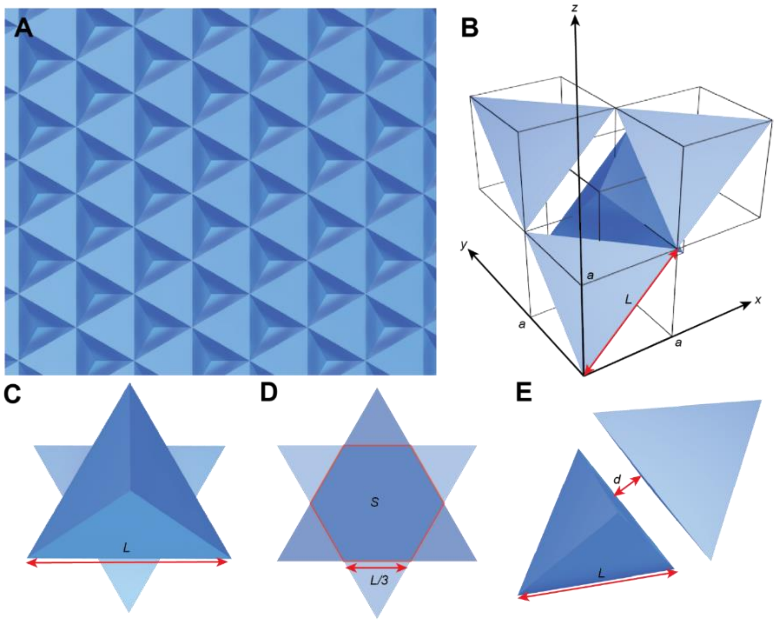

Supplementary Figure 1. Schematic illustration and 3D models of the achiral (1) structure. A) Extended lattice of achiral (1) assemblies; B) Model coordinate system used to calculate the contact area and interparticle distances; C) Projection of two tetrahedra NPs along their [111] axis and D) Overlap area *S* of two Td {111} planes; E) Interparticle distance between two tetrahedra NPs.

2.b. For achiral (2) assemblies: we considered a structure identical to achiral (1) in which the top layer of Td (tips pointing down, Supplementary Figure 2A below) are shifted along a unit vector such that each particle has one nearest neighbor (NN) with face-to-face spacing smaller than the d_{min} calculated above but two next-nearest neighbors (NNN) with spacing larger than d_{min}. Since the achiral (1) structure is unfavorable because of the geometric constraint imposed by the sharp tips that sets a large value for d_{min}, we hypothesized that this translation of the top layer of particles might result in a lower energy configuration. This structure maintains the tip-to-tip packing of achiral (1) with a hexagon-shaped NN contact area and a smaller elongated hexagonal parallelogram-shaped NNN contact area.

After shifting a layer of tetrahedra to a new NN particle distance of d^* (Supplementary Figure 2A, red arrow) a parameter Δd can be defined as:

$$\Delta d = d - d^* = \frac{\sqrt{6}}{12}L - d^* \qquad \text{(Supplementary eq. 6)}$$

This changed distance can be treated as the projection of movement of tetrahedra along the normal direction of their surface, as shown in Supplementary Figure 2B. Based on the structure geometry, this movement (M) is related to the dihedra angle (α), where in our case cos (α) = 1/3, and sin(α) = $2\sqrt{2}/3$):

$$M = \frac{\Delta d}{\sin a} = \frac{\Delta d}{2\sqrt{2}/3} = \frac{3\sqrt{2}}{4}\Delta d \qquad \text{(Supplementary eq. 7)}$$

After this movement, the distance (d') between the NNN facets is enlarged (Supplementary Figure 2A-2C), which can be calculated from the top view of the assemblies in Supplementary Figure 2D and 2E. The movement (M) is a vector and can be decomposed into vectors describing the movement along the tetrahedron face (M') and the movement perpendicular to the tetrahedron face (M'') given by:

$$M' = M\cos 30^o = \frac{\sqrt{3}}{2}M = \frac{3\sqrt{6}}{8}\Delta d \qquad \text{(Supplementary eq. 8)}$$

$$M'' = M\sin 30^o = \frac{1}{2}M = \frac{3\sqrt{2}}{8}\Delta d \qquad \text{(Supplementary eq. 9)}$$

The NNN interparticle distance (d') can be regarded as the original distance between two tetrahedra plus the movement distance:

$$d' = d + \frac{2\sqrt{2}}{3}M" = d + \frac{1}{2}\Delta d = \frac{\sqrt{6}}{12}L + \frac{1}{2}\left(\frac{\sqrt{6}}{12}L - d^*\right) = \frac{\sqrt{6}}{12}L - \frac{1}{2}d^* \qquad \text{(Supplementary eq. 10)}$$

The NN contact area maintains the same value (S) as achiral (1). However, the contact area of NNN faces (S') is reduced, as shown in Supplementary Figure 2E. The movement (M') along the tetrahedron face, as shown in Supplementary Figure 2F, can be used to calculate the new overlap area:

$$S' = \left(\frac{2}{3}L - \frac{3\sqrt{6}}{8}\Delta d\right) \times \frac{2}{3}L \times \frac{\sqrt{3}}{2} - 2 \times \frac{\sqrt{3}}{4}\left(\frac{L}{3} - \frac{3\sqrt{6}}{8}\Delta d\right)^2 = \frac{229\sqrt{3}}{1536}L^2 + \frac{27\sqrt{2}}{128}Ld^* - \frac{27\sqrt{2}}{64}d^{*2} \qquad \text{(Supplementary eq. 11)}$$

For the achiral (2) assemblies, the corresponding S, S' and d' are:

S = 1268.926 nm^2

S' = 1135.094 + 19.778d^*-0.731d^{*2} nm^2

d' = 20.300 – 0.5d^* nm

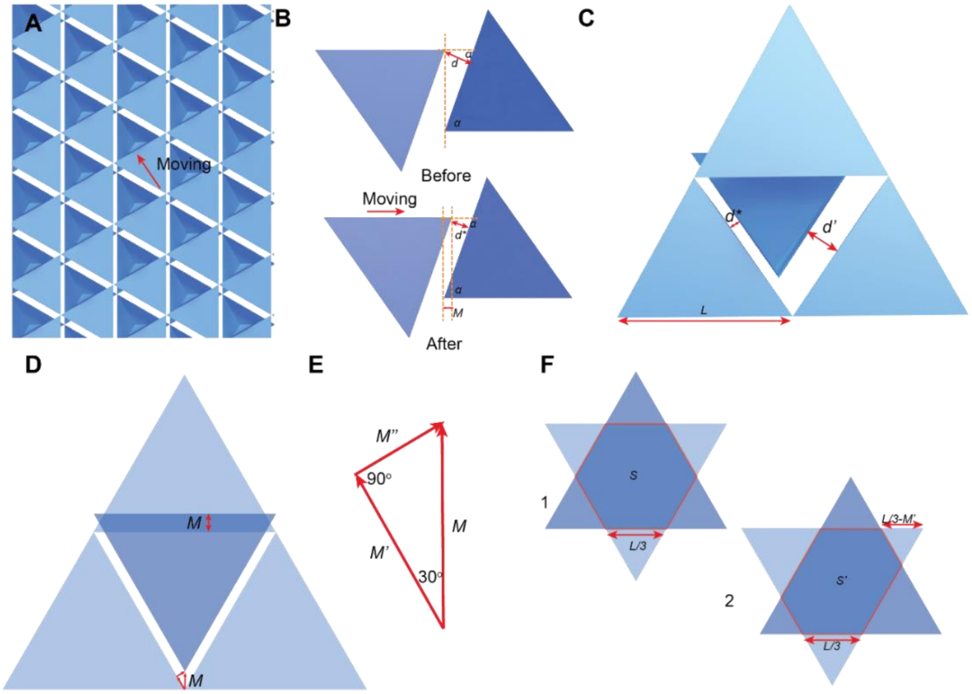

Supplementary Figure 2. Schematic illustration and 3D models of the achiral (2) structure. A) Extended lattice of achiral (2) assemblies, arrow indicates the top layer translation direction; B) Side view. The change in NN distance (*Δd*) can treated as the projection of movement along the normal of the tetrahedron plane; C) Projection showing the two types of interparticle distance. One is reduced (*d**) and the other is increased (*d'*) relative to achiral (1); D) Top view of the assembly with parameters used to calculate the contact area and NNN distance; the small red triangle is enlarged in Supplementary Figure 2E; E) Illustration of the relationship between the movement vectors (M, M', and M''); F) Two types of particle-particle contact area, (*S*) corresponding to NN (*d**) facets and (*S'*) corresponding to NNN facets (*d'*).

<u>2.c. For the packing of tetrahedra with rounded tips</u>: Chemically etching tetrahedra results in selective rounding of their tips (Supplementary Figure 3A, below), which results in a decreased minimum interparticle distance (Supplementary Figure 3B) while maintaining a large contacting area. We define the tip radius of curvature (R, Supplementary Figure 3C) and calculate the resulting minimum the interparticle distance (d^R_{min}) using (*H*), the projection of the particle-particle movement. Based on the geometrical model (Supplementary Figure 3C inset, 3D), *H* can be calculated from:

$$H = O'B - O'A = R - O'A \qquad \text{(Supplementary eq. 12)}$$

$$O'A = \frac{O'D}{\cos 30^o} = \frac{O'E - CD}{\cos 30^o} = \frac{O'E - AC}{\cos 30^o} = \frac{O'E - O'A\sin 60^o}{\cos 30^o} = \frac{R - \sqrt{3}R/2}{\sqrt{3}/2} = \frac{2\sqrt{3}-3}{3}R \qquad \text{(Supplementary eq. 13)}$$

H can be obtained:

$$H = R - O'A = R - \frac{2\sqrt{3}-3}{3}R = \frac{6-2\sqrt{3}}{3}R \qquad \text{(Supplementary eq. 14)}$$

The minimum interparticle distance as a function of tip rounding (d^R_{min}) is:

$$d^R_{min} = d_R = d - \frac{2\sqrt{2}}{3}H = \frac{\sqrt{6}}{12}L - \frac{2\sqrt{2}}{3}\frac{6-2\sqrt{3}}{3}R = \frac{\sqrt{6}}{12}L - \frac{12\sqrt{2}-4\sqrt{6}}{9}R \qquad \text{(Supplementary eq. 15)}$$

The contact area (S) is independent of tip radius and identical to that for achiral (1). For our Td particles with 66.3 nm edge length, the simplified expressions are:

$$S = 1268.926 \text{ nm}^2$$

$$d_{min}^{R} = 13.533 - 0.797R \text{ nm}$$

These equations were used to calculate the energetic stability of different achiral (1) structures as a function of tip radius, allowing for the mapping of the chiral (3) to achiral (1) phase transition (Figure 3g).

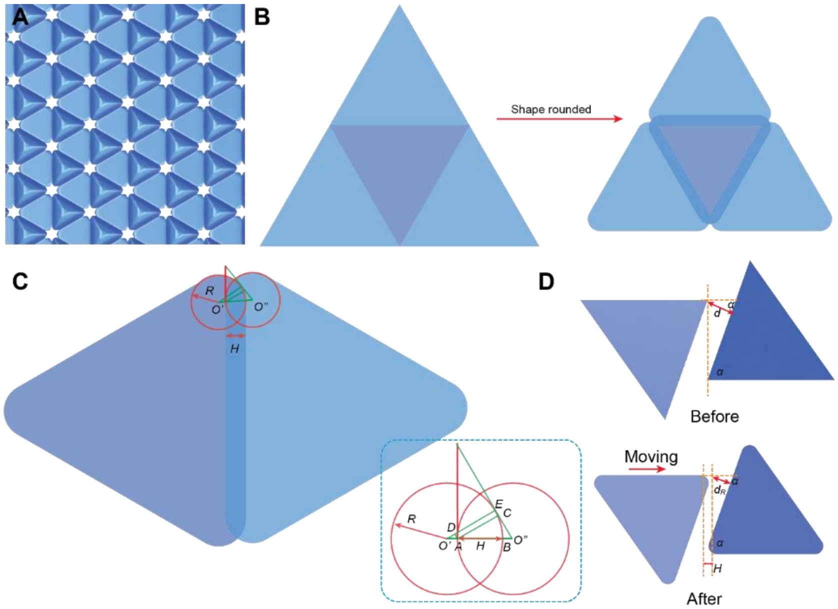

Supplementary Figure 3. Schematic illustration and geometric models for rounded tetrahedra assemblies. A) Extended lattice of achiral (1) assemblies of rounded Td; B) Increased radius of curvature in rounded Td results in decreased minimum interparticle distance, allowing for more dense packing; C) Top view of two rounded Td NPs with detailed geometrical relationships as inset; D) Side view of two Td NPs before and after tip rounding with decreased d_{min}^{R}.

<u>2.d. For the transition from achiral (1) to chiral (3) via rotation-based phase change:</u> We considered the possibility for a structure in the achiral (1) phase with sharp tips and large d_{min} to lower the energy of the system by rotating tetrahedra about their central [111] axis (Supplementary Figure 4A-4D, below) by an angle, θ (Supplementary Figure 4E). By avoiding tip-to-tip contact, this structure can have a lower d_{min} and more favorable interactions at the expense of lower face-to-face contact area and less favorable interactions. Both clockwise rotation ($0 < \theta < 30°$) and counterclockwise rotation ($0 > \theta > -30°$) result in degenerate energies, explaining the racemic mixture of planar chiral superlattice enantiomers. After rotation by an angle (θ), the minimum interparticle distance between two tetrahedra is denoted d_{min}^{θ} and the contact area is denoted S'' (Supplementary Figure 4D, 4G-4I).

As shown in Supplementary Figure 4E, we define R_0 and R_1 as the radius of circumscribed circles in the middle of an equilateral triangular Td face before and after rotation, respectively. The corresponding relationship between the offset distance (D) and rotation angle (θ) can be obtained by the enlarged triangle in blue (Supplementary Figure 4F), which is:

$$h = L\sin\theta \qquad \text{(Supplementary eq. 16)}$$

$$D = \frac{h}{\sin(\theta + 60^o)} \qquad \text{(Supplementary eq. 17)}$$

Therefore,

$$D = \frac{L\sin\theta}{\sin(\theta + 60^o)} \qquad \text{(Supplementary eq. 18)}$$

The offset distance (*D*) and the edge length of tetrahedra (*L*) can be measured from SEM images of chiral (3) assemblies (Figure 4d), allowing for calculation of the experimental rotation degree (*θ*) from Supplementary eq. 19 as:

$$\theta = \arctan\left(\frac{\sqrt{3}D}{2L - D}\right) \qquad \text{(Supplementary eq. 19)}$$

Then, based on the enlarged triangle in green (Supplementary Figure 4F), the relationship between R_0, R_1, *D'* and *D"* can be obtained:

$$R_0 = \frac{\sqrt{3}L}{3}$$

$$R_1 = \frac{\sqrt{3}}{3}(L - D)$$

$$R_0 - R_1 = \frac{\sqrt{3}D}{3} = \frac{\sqrt{3}L\sin\theta}{3\sin(\theta + 60^o)}$$

$$D' = (R_0 - R_1)\sin(30^o) = \frac{R_0 - R_1}{2} = \frac{\sqrt{3}L\sin\theta}{6\sin(\theta + 60^o)} \qquad \text{(Supplementary eq. 20)}$$

$$D^" = (R_0 - R_1)\cos(30^o) = \frac{\sqrt{3}(R_0 - R_1)}{2} = \frac{L\sin\theta}{2\sin(\theta + 60^o)} \qquad \text{(Supplementary eq. 21)}$$

Similar to the movement in the achiral (2) case, the offset distance (*D*) can be used to calculate the minimum particle distance from the movement perpendicular to the tetrahedron face (*D'*) as shown in Supplementary Figure 4G and H:

$$d_{min}^{\theta} = d" = d - \frac{2\sqrt{2}}{3}D' = \frac{\sqrt{6}}{12}L - \frac{\sqrt{6}L\sin\theta}{9\sin(\theta + 60^o)} \qquad \text{(Supplementary eq. 22)}$$

The decreased contact area (*S"*) can be calculated based on Supplementary Figure 4I:

$$S" = \frac{\sqrt{3}}{6}L^2 - \frac{\sqrt{3}}{8}L^2\frac{\sin^2\theta}{\sin^2(\theta + 60^o)} \qquad \text{(Supplementary eq. 23)}$$

For the chiral (3) assemblies in this work, the offset distance (*D*) is 24.9 nm. Based on Supplementary eq. 19, 22 and 23, the corresponding parameters are:

θ =21.8285^o

d_{min} = *d"* = 6.892 nm

S" = 1134.634 nm^2

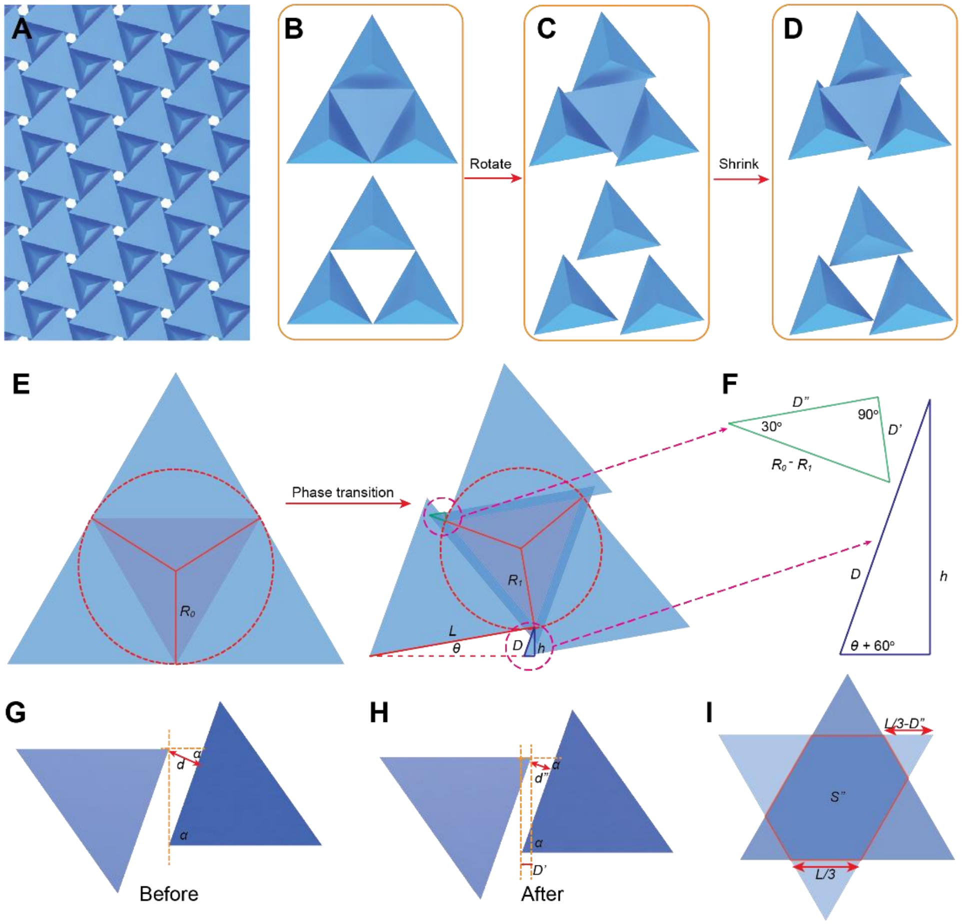

Supplementary Figure 4. Schematic illustration and geometric models for rotation phase transformation. A) Extended lattice of chiral (3) assemblies; B, C, D) Phase transformation for tetrahedra assemblies from achiral (1) to chiral (3) via Td rotation and lattice contraction; bottom images omit the central Td particle for clarity; E) Top view and geometric models of lattices before and after rotation; the small green and blue triangles are enlarged in Supplementary Figure 4F; F) Geometric relationships between offset vectors *D, D', and D''*; G) Interparticle distance between Td in achiral (1) structure, where α and d represent the dihedral angle and the interparticle distance, respectively; H) Interparticle distance between Td in chiral (3) structure, where D' is the in-plane movement and causes the reduced minimum interparticle distance (d^{θ}_{min}); I) Face-to-face contact area after rotation transition (*S''*), where *D''* is the movement along the tetrahedra.

Supplementary Figures

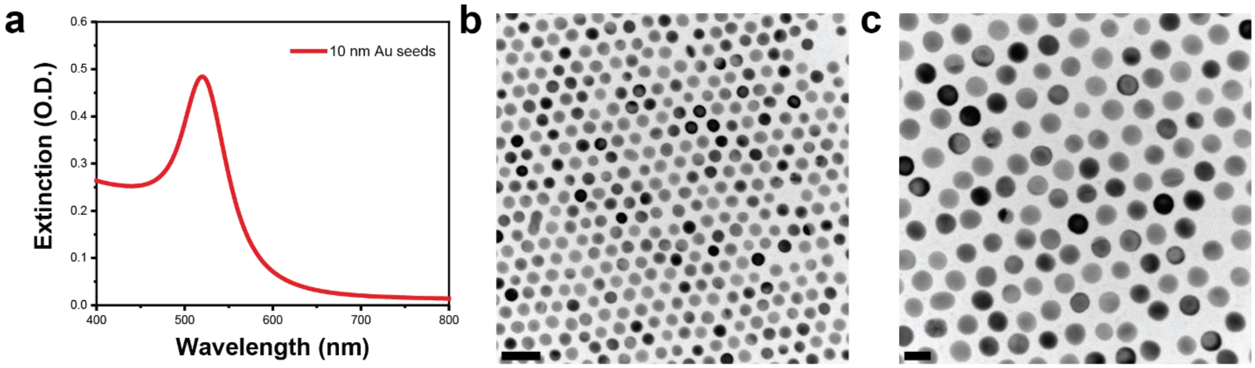

Supplementary Figure 5. 10 nm Au seeds for tetrahedra syntheses. a) UV-vis spectrum of 10 nm Au seeds; b) TEM image of uniform 10 nm Au single crystalline seeds with purity around 85%; c) Magnified TEM image shows the existence of twinned impurities. Scale bars: b) 50 nm; c) 20 nm.

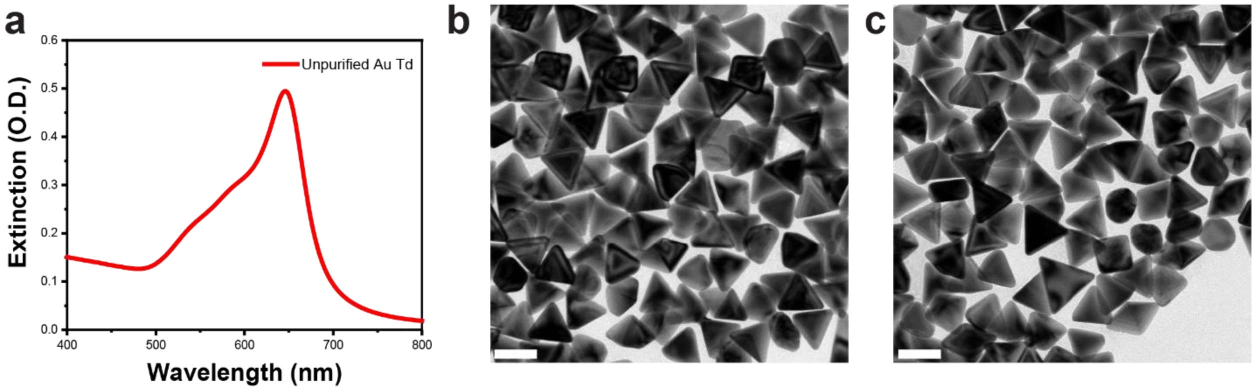

Supplementary Figure 6. As-synthesized Au Td NPs from 10 nm Au seeds. a) The UV-vis spectrum of the unpurified Au Td NPs; b and c) TEM images of Au Td NPs with impurities. All scale bars are 50 nm.

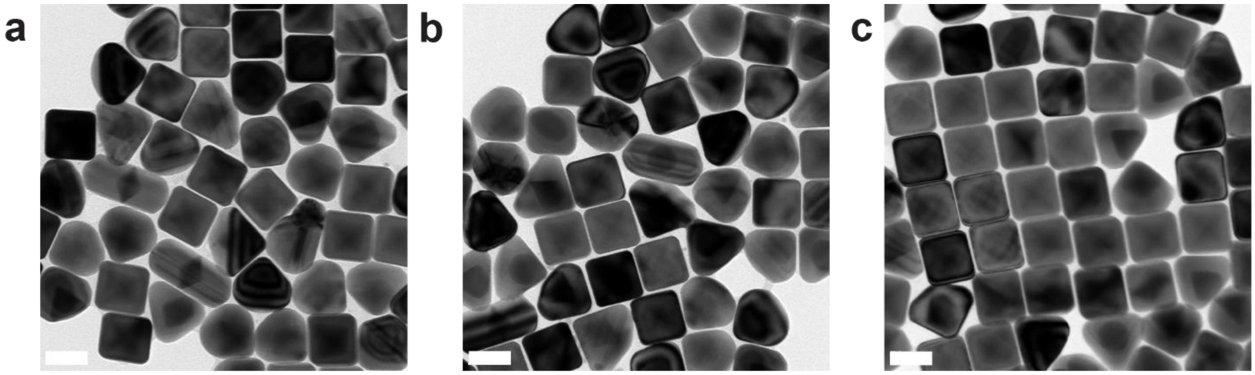

Supplementary Figure 7. TEM images of the as-synthesized core-shell Au@Ag NPs. Au Td@Ag cube NPs, Au decahedra @Ag Nanorods, and Au bitetrahedra@Ag Right Bipyramid NPs along with other impurities can be seen in (a), (b), and (c). All scale bars are 50 nm.

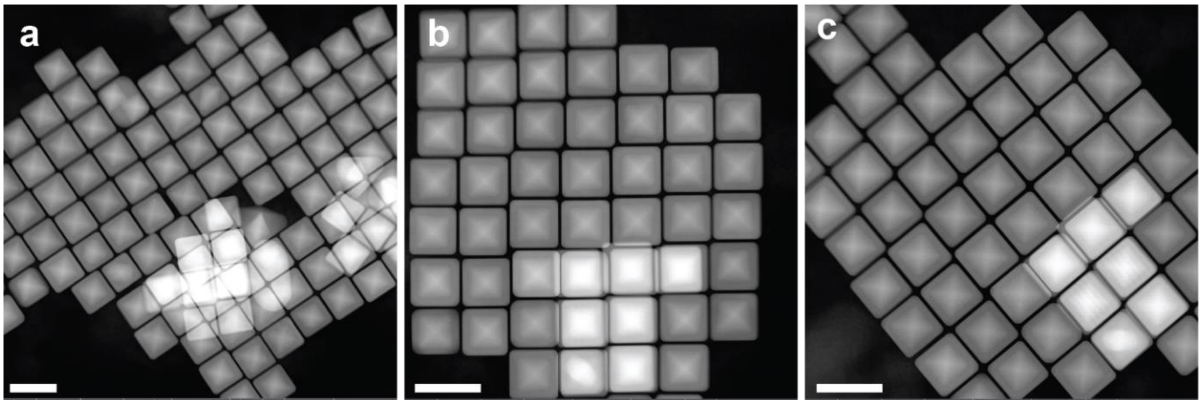

Supplementary Figure 8. (a-c) HAADF-STEM images of purified core-shell Au Td@Ag cube NPs. All scale bars are 100 nm.

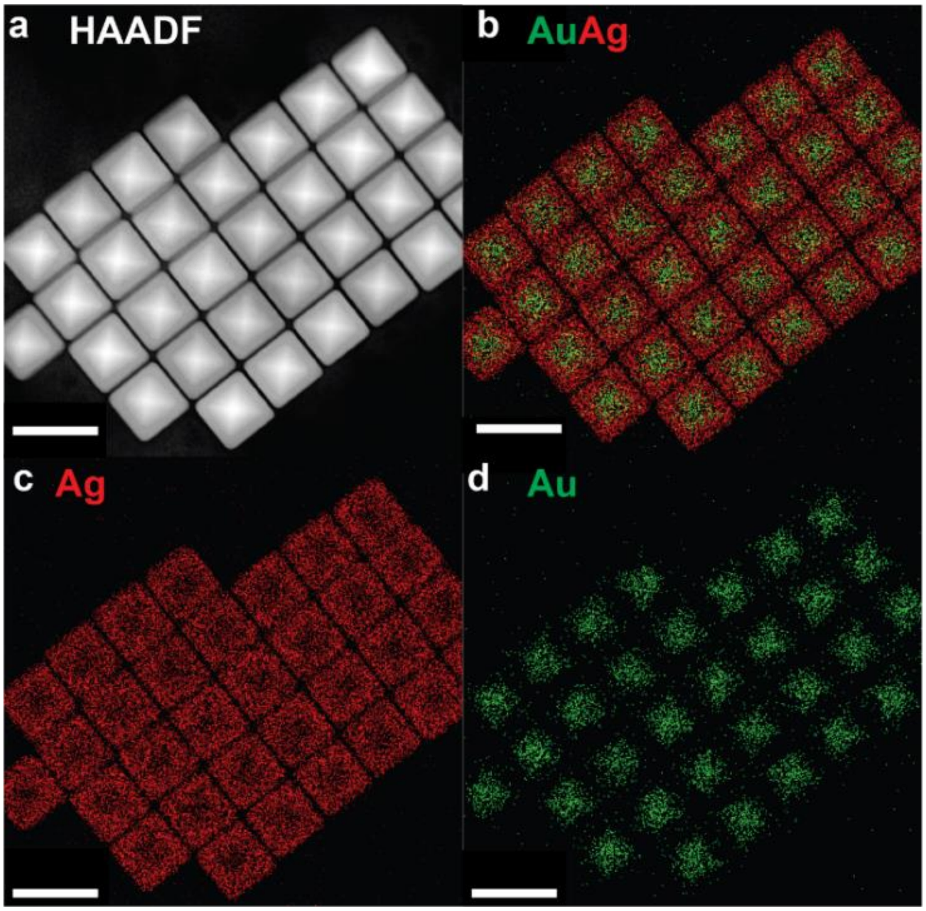

Supplementary Figure 9. EDS mapping of core-shell Au Td@Ag cube NPs. a) HAADF-STEM image; b) Overlapped Au and Ag elemental maps; c) mapping of Ag cube shell; d) mapping of Au Td core. All scale bars are 100 nm.

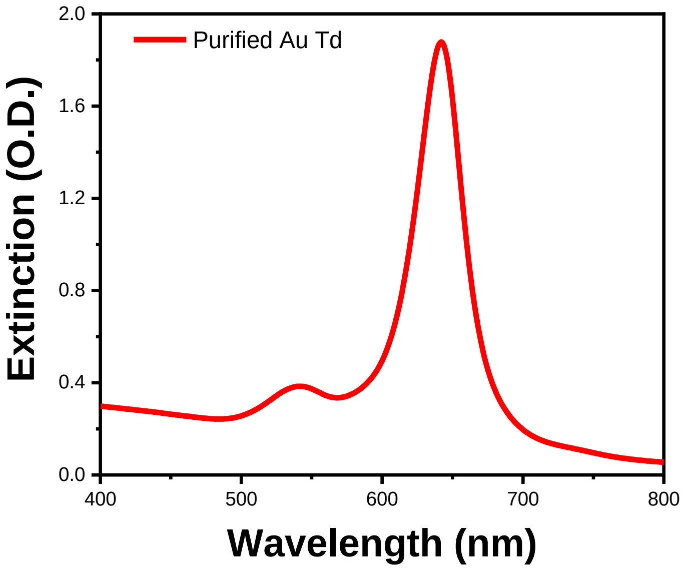

Supplementary Figure 10. UV-vis of purified Au Td NPs. Unpurified Au Td NPs have a broad shoulder on the blue side of the dipole plasmon (Fig. S2) which resolves into a distinct peak located at ~541 nm after separation, demonstrating the improved purity.

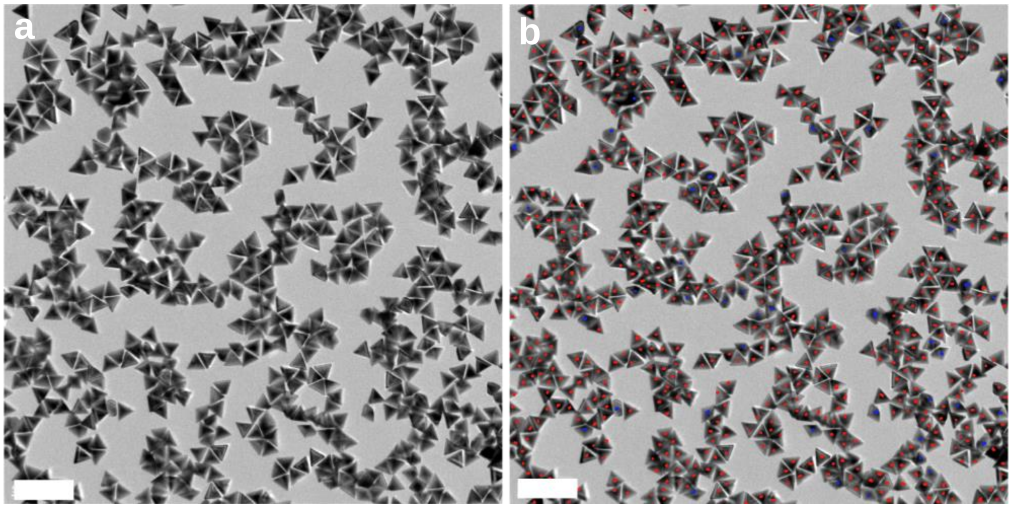

Supplementary Figure 11. Analysis of the purity of Au Td NPs from TEM images. Au Td NPs were first deposited on TEM grids by fast evaporation to make them disperse evenly, after which a series of (a) TEM images were taken from different areas to sample a representative population. (b) Red and blue dots were assigned to particles based on whether they belong to Td or impurity categories, respectively. More than 17,000 particles were counted by this method to determine the purity of separated Au Td samples. All scale bars are all 200 nm.

Supplementary Table 1: Analysis of purity of Au Td NPs before and after separation

Image	After purification			As synthesized		
	Td NPs	Impurities	Purity (%)	Td NPs	Impurities	Purity (%)
1	660	30	95.652	458	67	87.238
2	650	24	96.439	668	43	93.952
3	746	33	95.764	362	256	58.576
4	721	45	94.125	465	201	69.820
5	683	45	93.819	522	215	70.828
6	768	48	94.118	438	181	70.759
7	728	37	95.163	654	268	70.933
8	604	28	95.570	408	211	65.913
9	473	26	94.790	215	65	76.786
10	710	36	95.174	656	242	73.051
11	690	30	95.833	538	285	65.371
12	680	40	94.444	506	209	70.769
13	736	31	95.958	568	230	71.178
14	738	30	96.093	603	203	74.814
15	672	42	94.118	551	283	66.067
**Total**	**10259**	**525**	**95.132**	**7612**	**2959**	**72.008**

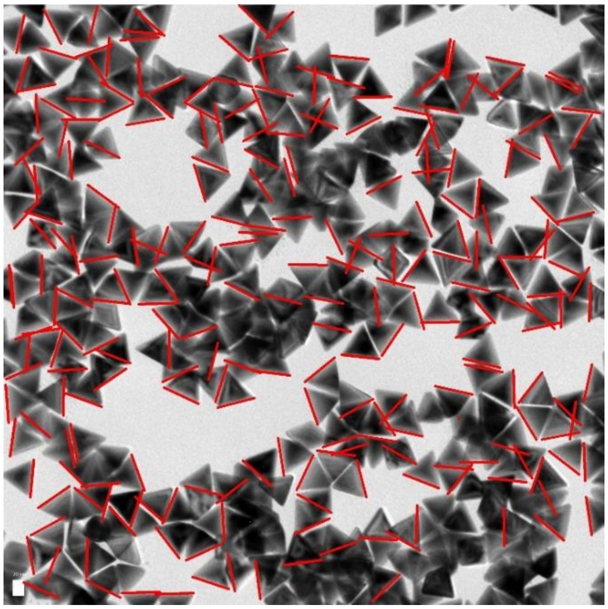

Supplementary Figure 12. Representative image used for the analysis of the size distribution of Au Td NPs after purification. More than 200 measurements were used in the calculation of statistical values.

Supplementary Tables 2: Dataset of size measurements of Au Td NPs after separation (edge length: nm)

63.59	63.55	63.53	63.89	63.61	64.42	64.24	64.17	64.51	64.25
63.64	62.61	64.41	64.69	65.11	64.54	64.29	64.46	65.42	64.32
63.65	63.29	65.29	65.4	65.13	65.35	65.26	65.46	65.44	65.23
65.21	63.42	65.29	65.5	65.13	65.41	65.4	65.55	65.64	66.12
65.23	63.57	65.35	66.12	66.16	66.13	66.23	66.13	66.22	66.16
66.04	63.68	65.37	66.15	66.19	66.15	66.23	66.22	66.23	66.16
66.23	64.27	65.41	66.22	66.23	66.23	66.24	66.23	66.24	66.19
66.23	64.32	66.15	66.25	66.23	66.26	66.26	66.25	66.25	66.21
66.29	64.35	66.24	66.26	66.24	66.32	66.3	66.25	66.28	66.23
66.35	65.26	66.26	66.26	66.24	66.32	66.31	66.26	66.28	66.23
66.49	65.44	66.26	66.29	66.26	66.39	66.33	66.26	65.29	66.24
66.64	66.26	66.26	66.3	66.26	66.44	66.35	66.26	66.29	66.26
67.24	66.26	66.26	66.35	66.26	66.48	66.35	66.26	66.31	66.26
67.37	66.3	66.33	66.37	66.26	66.48	66.35	66.27	66.31	66.27
67.54	66.32	66.35	66.38	66.26	66.56	66.37	66.31	66.35	66.28
67.15	66.33	66.36	66.39	66.26	66.56	66.38	66.32	66.35	66.28
68.18	66.38	66.38	66.49	66.28	66.58	66.38	66.35	66.38	66.29
68.3	66.43	66.4	66.52	66.28	66.59	66.38	66.35	66.42	66.31
68.48	67.12	66.45	66.53	66.29	66.62	66.42	66.35	66.47	66.32
68.5	67.44	66.51	66.55	66.3	66.63	66.43	66.43	66.56	66.33
68.53	67.23	67.26	66.56	66.31	66.68	66.47	66.64	66.57	66.35
68.65	68.42	67.51	66.58	66.51	67.24	66.54	66.67	67.29	66.35
68.65	68.43	68.22	67.17	67.32	67.51	67.46	67.41	68.21	67.41
68.67	68.62	68.38	68.24	67.35	68.25	67.51	68.36	68.4	68.22
68.68	69.02	68.61	68.71	68.13	68.62	67.51	67.81	68.51	68.24
**Average**	66.3039	**Standard deviation**			1.14	**Coefficient of variation**			1.72%

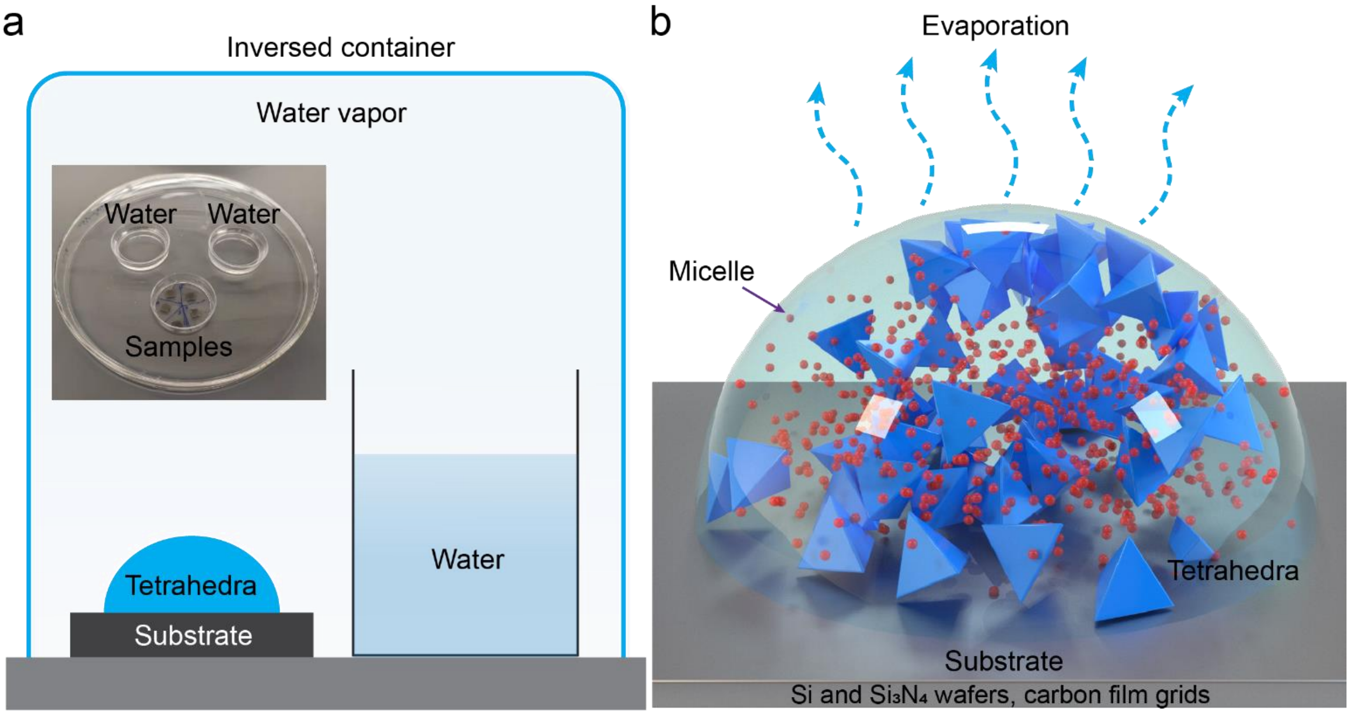

Supplementary Figure 13. Setup for the self-assembly of tetrahedra on a substrate. a) Au Td NPs assembled on substrates in high humidity allows for control of the evaporation rate, inset is a photo of the experimental setup; b) Schematic illustration of the assembly process of Au Td NPs during evaporation. Over time, the concentration of Au Td NPs, electrolytes, and micelles increases, creating a balance of forces which drives 2D assembly on the substrate.

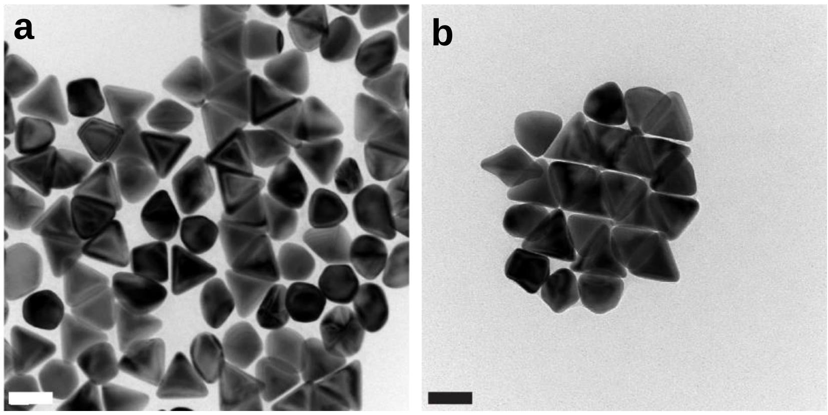

Supplementary Figure 14. (a, b) TEM images of impure Au Td NPs assembled via slow solvent evaporation (Supplementary Figure 13), resulting in limited packing behavior and short-range order. All scale bars are 50 nm.

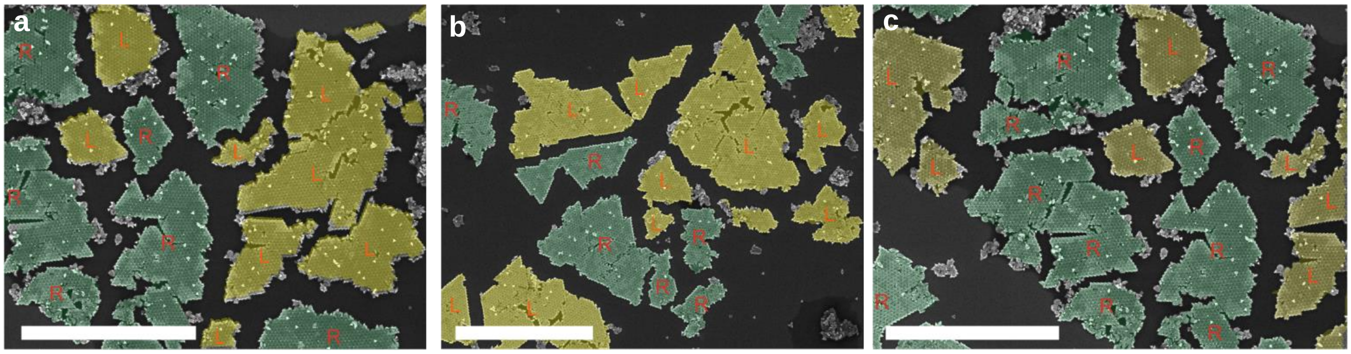

Supplementary Figure 15. (a-c) False-colored low magnification SEM images of Au Td assemblies with R/L planar chiral handedness denoted by green and yellow shading, respectively. Averaged over the entire sample, R/L domains are present in similar number and are of similar size, as expected. All scale bars are 3 μm.

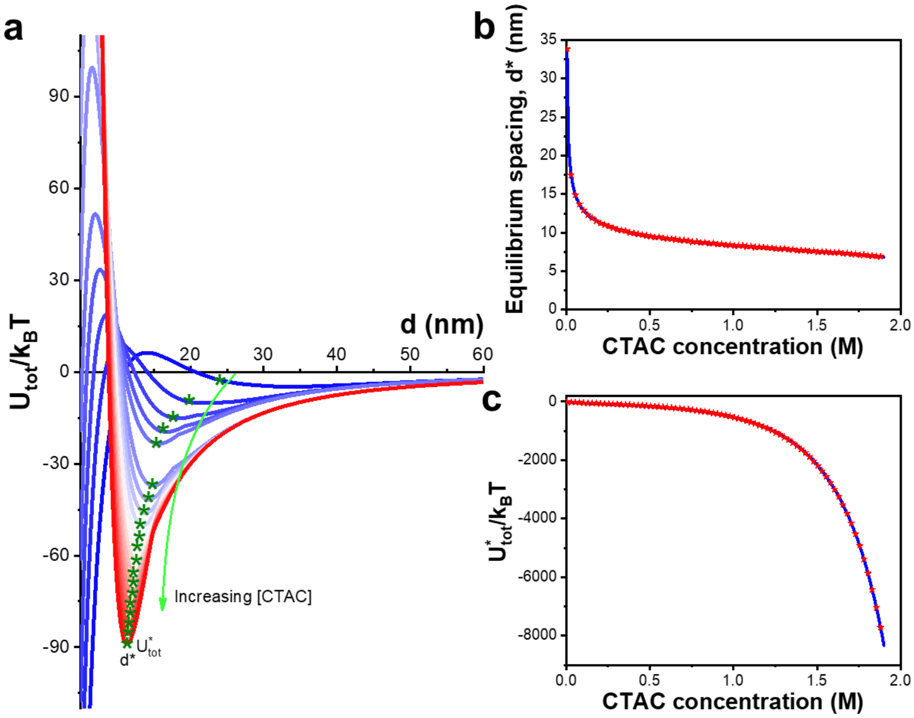

Supplementary Figure 16. Method for calculating plots of U_{tot}* vs. CTAC concentration. a) First, total interaction potential (U_{tot}, which is the sum of van der Waals, electrostatic, and depletion forces for the overlap of all neighboring Td faces in a superlattice) is calculated as a function of interparticle separation (*d*). The position of the local minimum of this function indicates the equilibrium particle spacing (*d**) and interaction strength (U_{tot}*). This calculation is then repeated for a range of increasing CTAC concentrations, which generally causes particle faces to pack more closely (b) and with stronger (c, more negative U_{tot}*) attraction. While plots of *d** vs. CTAC show very little dependence on the superlattice configuration, U_{tot}*vs. CTAC is highly dependent on the facet overlap area experienced between neighboring Td in an assembly and thus can be used to identify the phase behavior of the system; more negative values of U_{tot}* at a given CTAC concentration indicate a more stable superlattice.

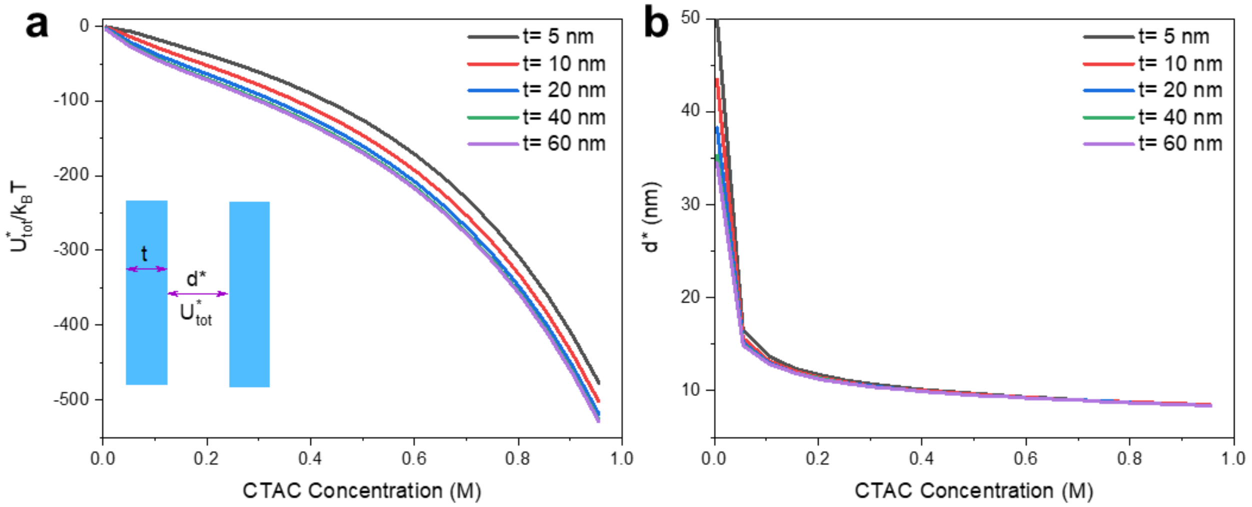

Supplementary Figure 17. Comparison of the equilibrium (a) interaction energy (U_{tot}*) and (b) interparticle spacing (*d**) for particles of finite thickness (*t*). Of all the forces involved in Td colloidal interactions, vdW is the only one dependent on a volume element, as opposed to a surface element, and therefore is the only one that will change as a function of particle thickness. Since the contribution of the van der Waals attraction to the overall interaction energy is accurately captured by considering only the first 5-10 nm of particle depth, we conclude that approximating the total particle interaction as a sum of 4 independent Td facet interactions with infinite depth is a reasonable simplifying assumption that captures the relevant system behavior (see methods section).

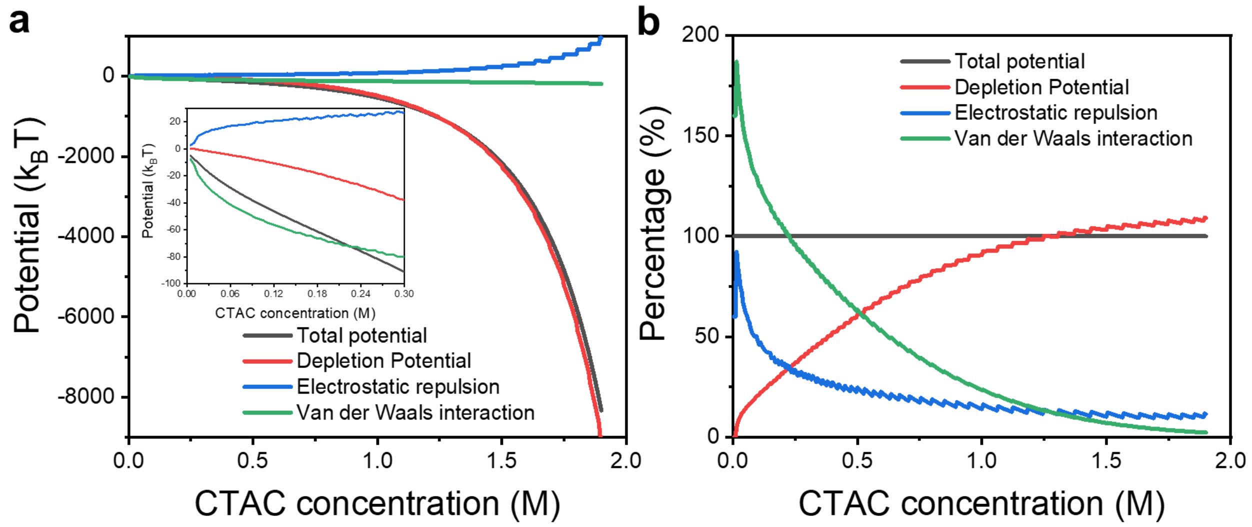

Supplementary Figure 18. Breakdown of the different contributions to the overall interaction potential involved in the assembly of Au Td NPs as function of CTAC concentration shown as absolute values (a) and percentage of total (b). van der Waals interactions dominate at low CTAC concentration while depletion attraction dominates at high CTAC concentration.

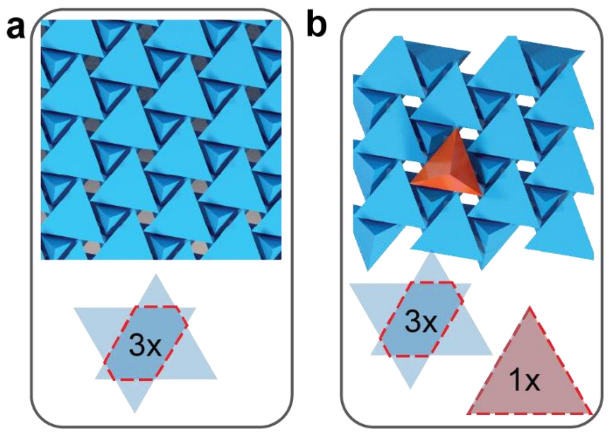

Supplementary Figure 19. Comparison of the facet overlap areas for a single Td particle in a (a) 2D and (b) 3D hexagonal chiral superlattice. For the 2D phase, each particle has three elongated hexagonal parallelogram-shaped overlaps while for 3D lattice, each particle has three elongated hexagonal parallelogram-shaped overlaps and one triangular overlap. These overlap areas are used to calculate and compare U_{tot}^{*} for 2D vs. 3D superlattices.

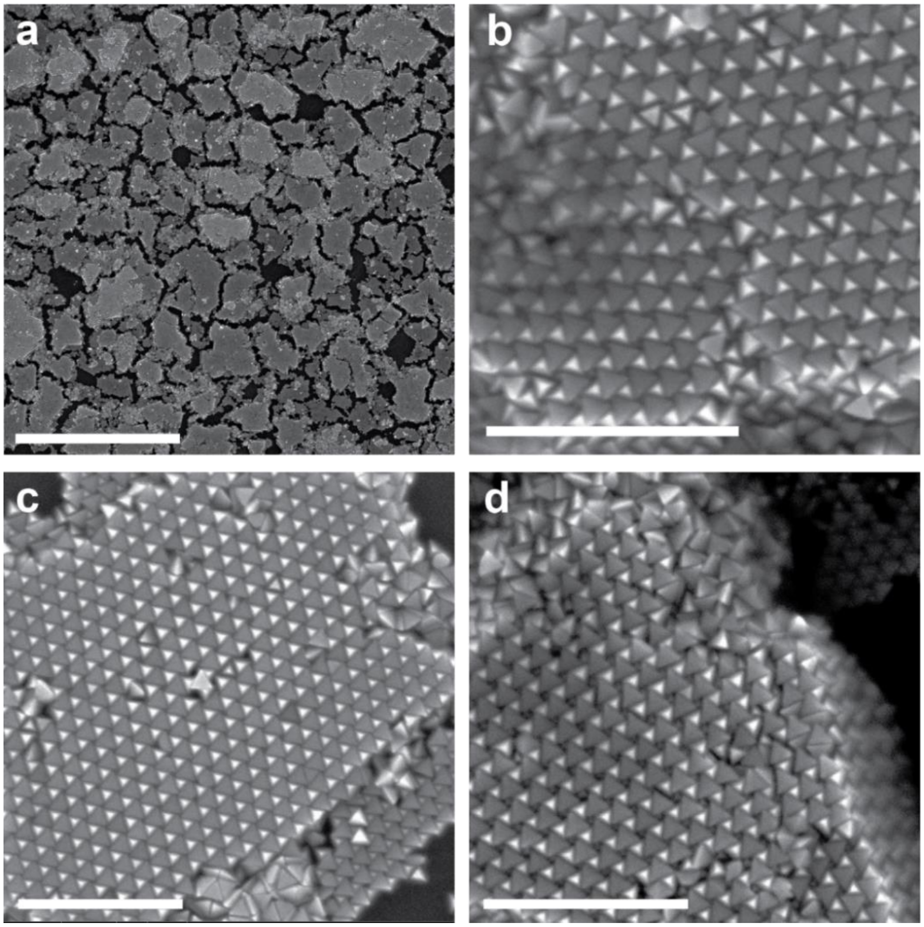

Supplementary Figure 20. (a) Low magnification and (b-d) high magnification images of superlattices that are the 3D analogue of the 2D chiral hexagonal phase, achieved by extremely slow evaporation of water during the assembly process. These achiral structures can be thought of as alternating stacks of left- and right-handed planar chiral superlattices. Scale bars: a) 5 μm; b, c and d) 500 nm.

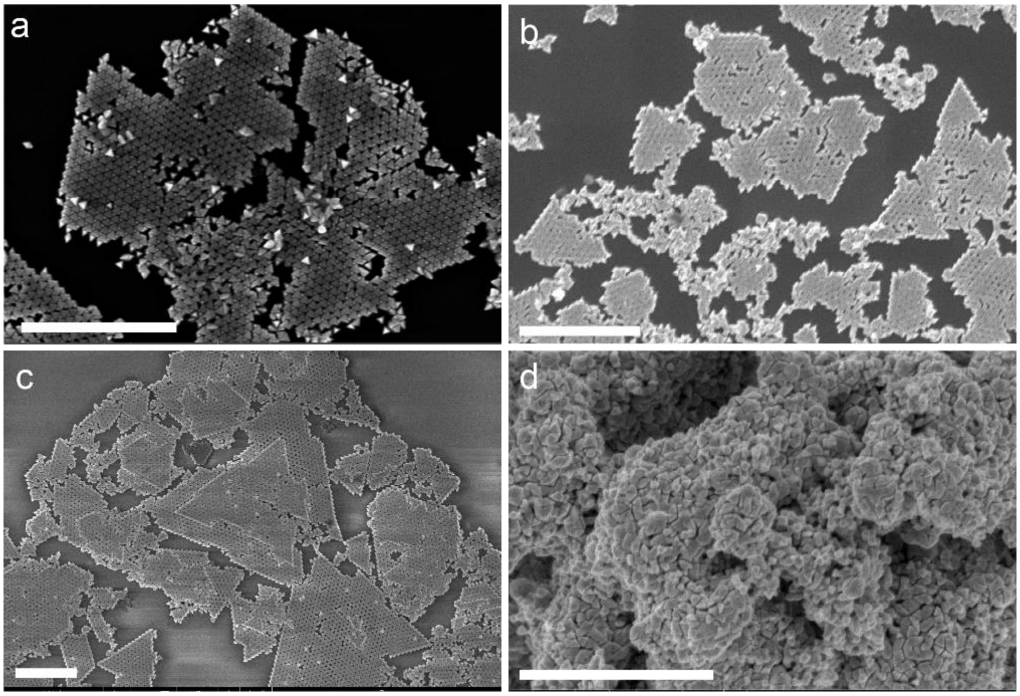

Supplementary Figure 21. Typical Au Td assemblies formed on the a) Si_3N_4; b) carbon; c) mica substrate and d) in the absence of substrate (precipitated from solution) with same assembly conditions. All scale bars are 1 μm.

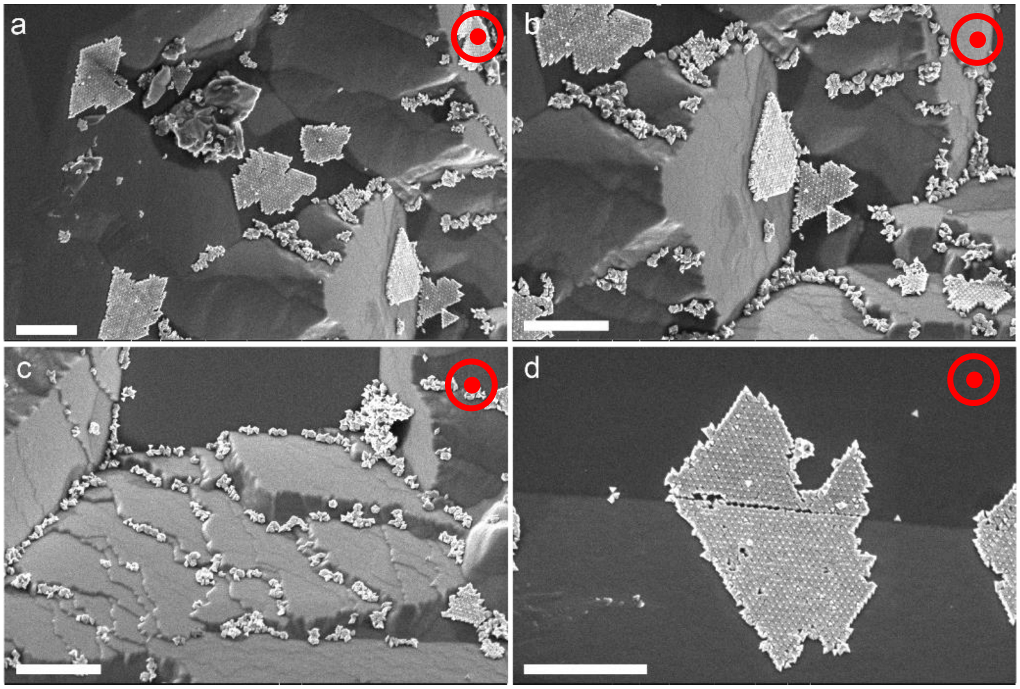

Supplementary Figure 22. (a-d) The backside of a silicon wafer, which presents a jagged array of crystalline steps with a variety of orientations, nonetheless still supports the formation of chiral Td superlattices. The direction of gravitational forces is into the plane of the image (denoted in red), indicating that the presence of a flat Si surface is all that is necessary to facilitate nucleation of 2D superlattices. Consequently, we conclude that sedimentation forces do not play a significant role in the assembly mechanism. All scale bars are 1 μm.

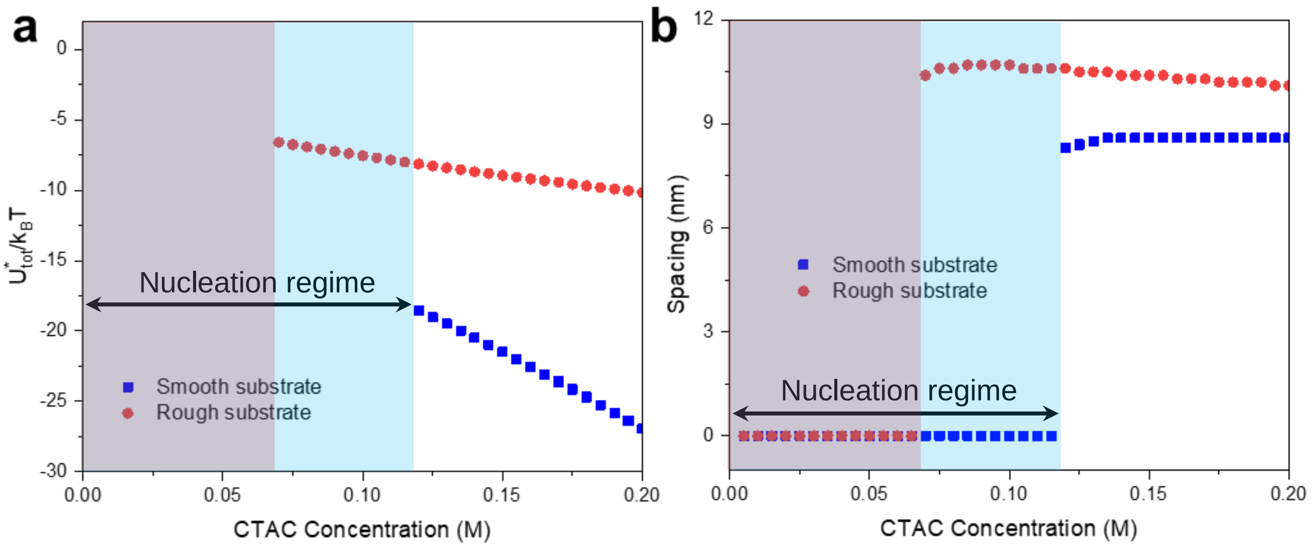

Supplementary Figure 23. Model calculations of the potential minimum, *Utot**, (a) and equilibrium spacing, *d**, (b) for the Td-substrate interaction alone. At low CTAC concentrations, Utot* values go to negative infinity and indicate that attractive substrate-particle interactions overwhelm weak electrostatic repulsions, resulting in a d* ≈ 0. However, at intermediate CTAC concentrations > ~100 mM, depletion interactions, whose length scale is set by the size of the CTAC micelle ~5 – 10 nm, become significant and can compete with vdW and electrostatic forces. The result is that the net interaction adopts a local minimum value, such that Utot* is well-defined (a), and Td rise off the surface with d* ≈ 9 nm (b). We propose that the range of CTAC concentrations for which Td are strongly-bound to the substrate (0 – 100 mM) is a "nucleation regime" where particles are drawn out of solution and concentrated at substrates, which facilitates the crystallization of 2D superlattice phases. If depletion forces are weakened to 10% of their original value (red data), which is consistent with rough surfaces, then the CTAC concentration at which Td rise off the substrate is lowered. This means that less time is spent in the nucleation regime during droplet evaporation and interparticle interactions are weaker during that time, both of which suggest fewer superlattice nuclei and/or more disordered assemblies. Also note that for rough substrates, when Td rise off the surface, Utot* is only ~6 kT, which may too weak to hold particles to the interface for long enough to allow 2D assembly to occur. Thus, substrates appear to act as nucleation sites for Td superlattices which, in addition to their kinetically preferred growth behavior, strongly favors 2D crystals.

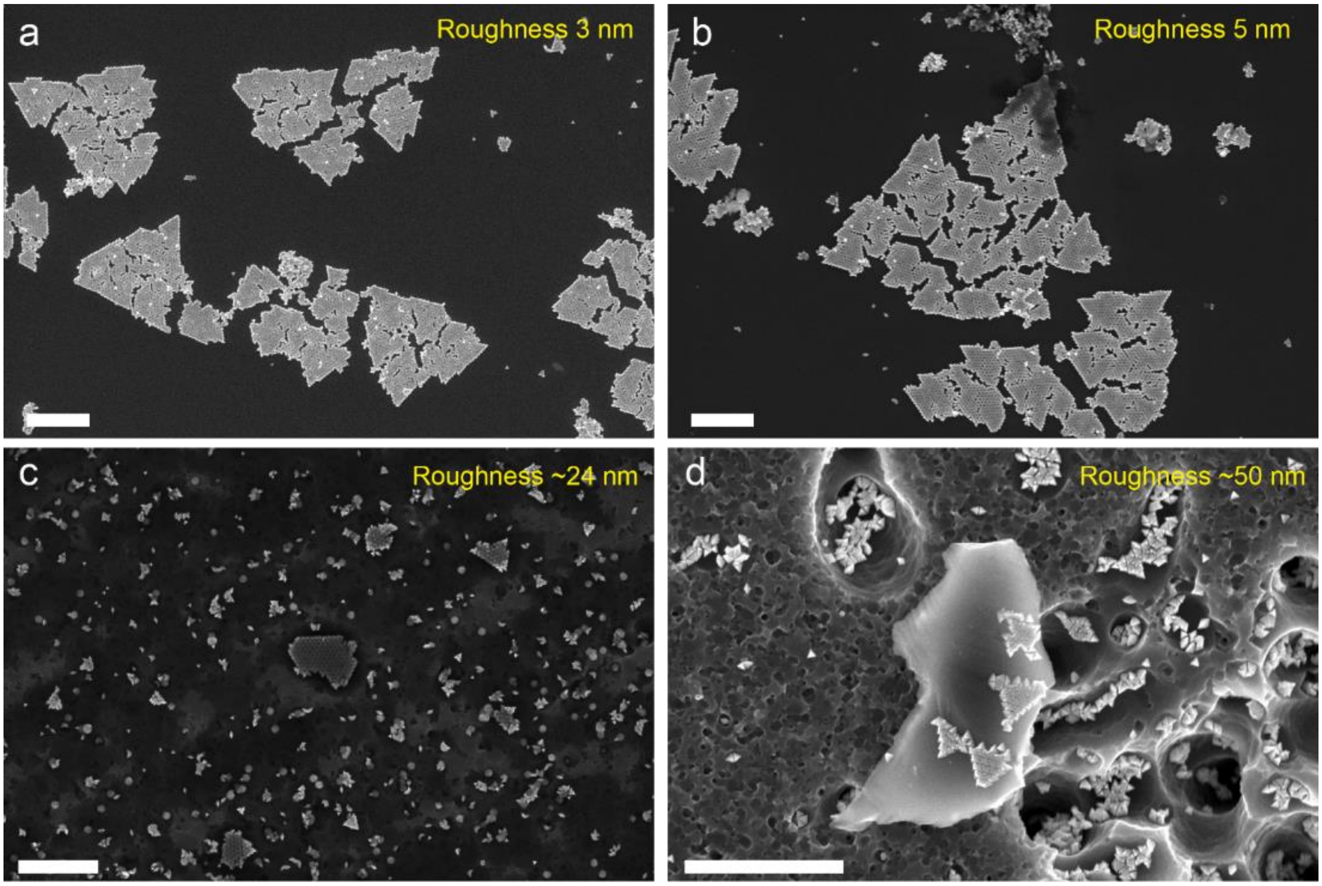

Supplementary Figure 24. The role of substrate surface roughness on the ability for 2D superlattices to form. As-obtained Si wafers are reported to have ~3 nm surface roughness, which was increased via reactive ion etching (RIE) plasma treatment (power of 100 W) with CF_4 (10 sccm) and oxygen (5 sccm) mixed gas with different times, a) 0 min; b) 1 min; c) 2 min and d) 5 min; resulting in substrates with measured surface roughness values of 3, 5, 24, and 50 nm, respectively. The reduced order and domain size with increased surface roughness is consistent with the importance of depletion forces in facilitating superlattice nucleation. All scale bars are 1 μm.

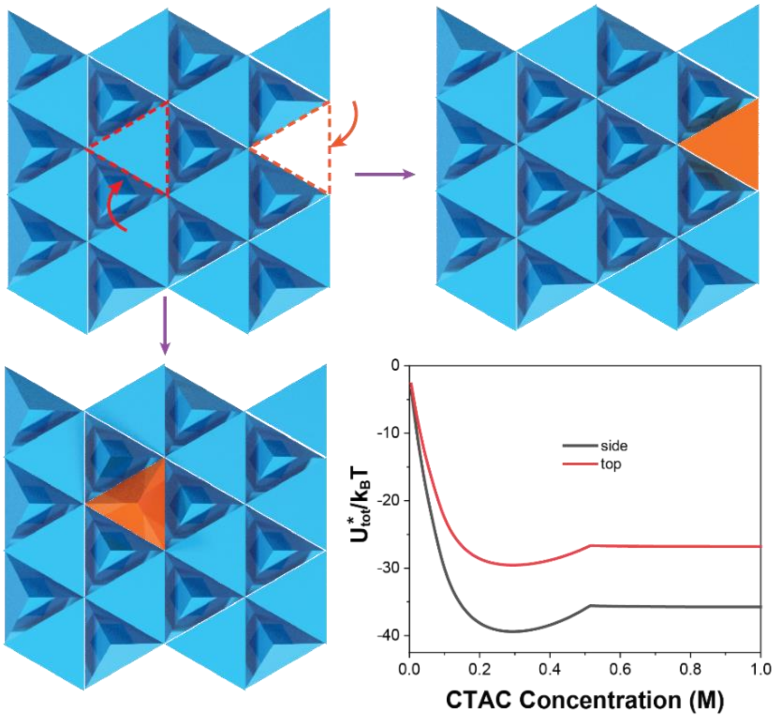

Supplementary Figure 25. Schematic and calculation of the stability of tetrahedron at top or side positions for the achiral (1) structure, demonstrating kinetically-preferred lateral growth. Asymptotic behavior arises because of a d_{min} that prevents further densification of Td at higher CTAC concentrations.

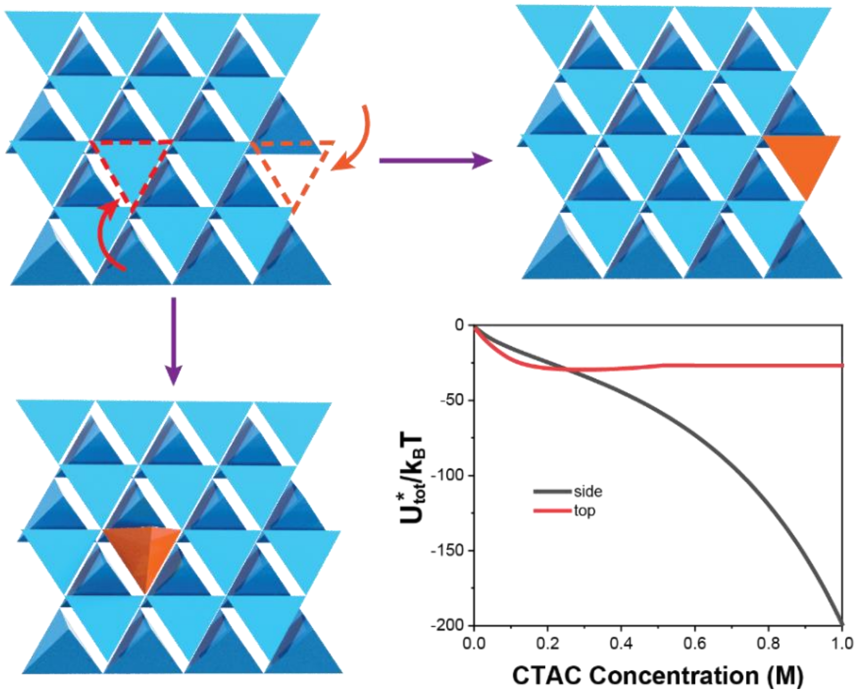

Supplementary Figure 26. Schematic and calculation of the stability of tetrahedron at top or side positions for the achiral (2) structure, demonstrating kinetically-preferred lateral growth. Asymptotic behavior arises because of a d_{min} that prevents further densification of Td at higher CTAC concentrations.

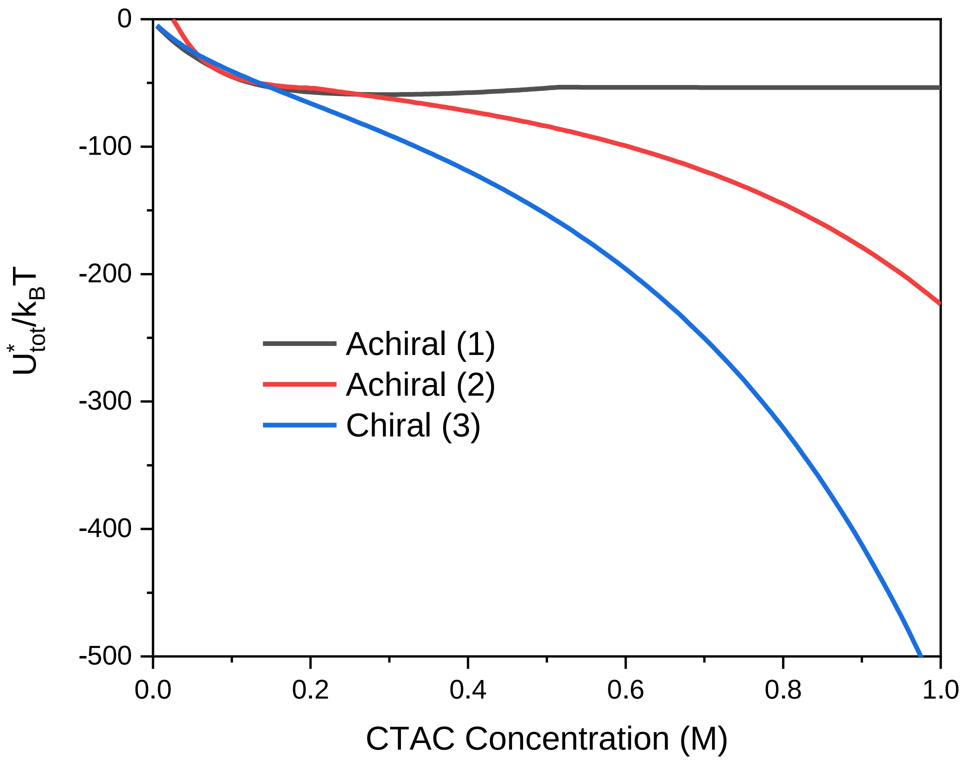

Supplementary Figure 27. Calculation of total energy vs. CTAC concentration for three different 2D assemblies of Td particles. Dataset is the same as Fig. 3d but plotted over a larger range of values.

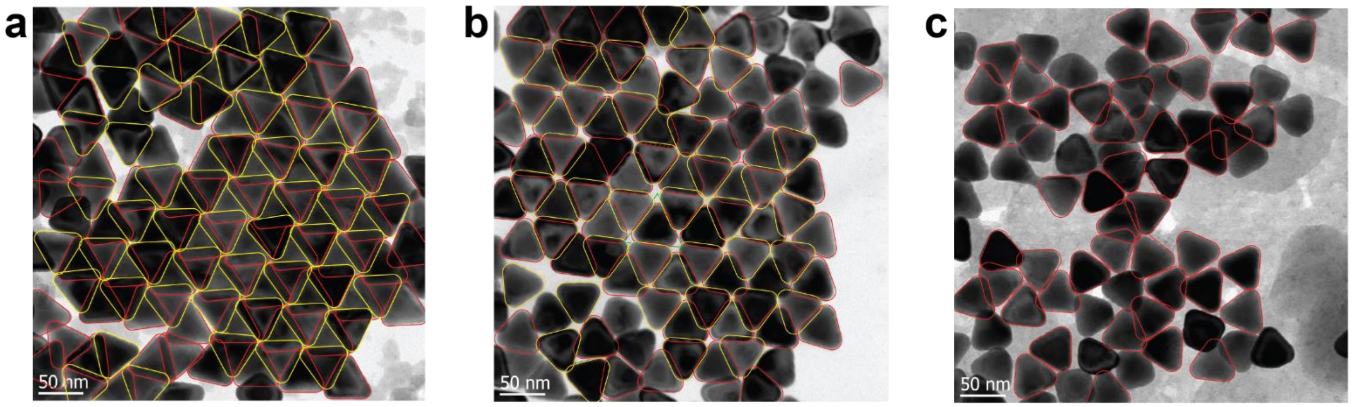

Supplementary Figure 28. Analysis of the assembly of tetrahedra with tips rounded to radius of curvature values of a) 7.5 nm, b) 8.4 nm and c) 11.9 nm.

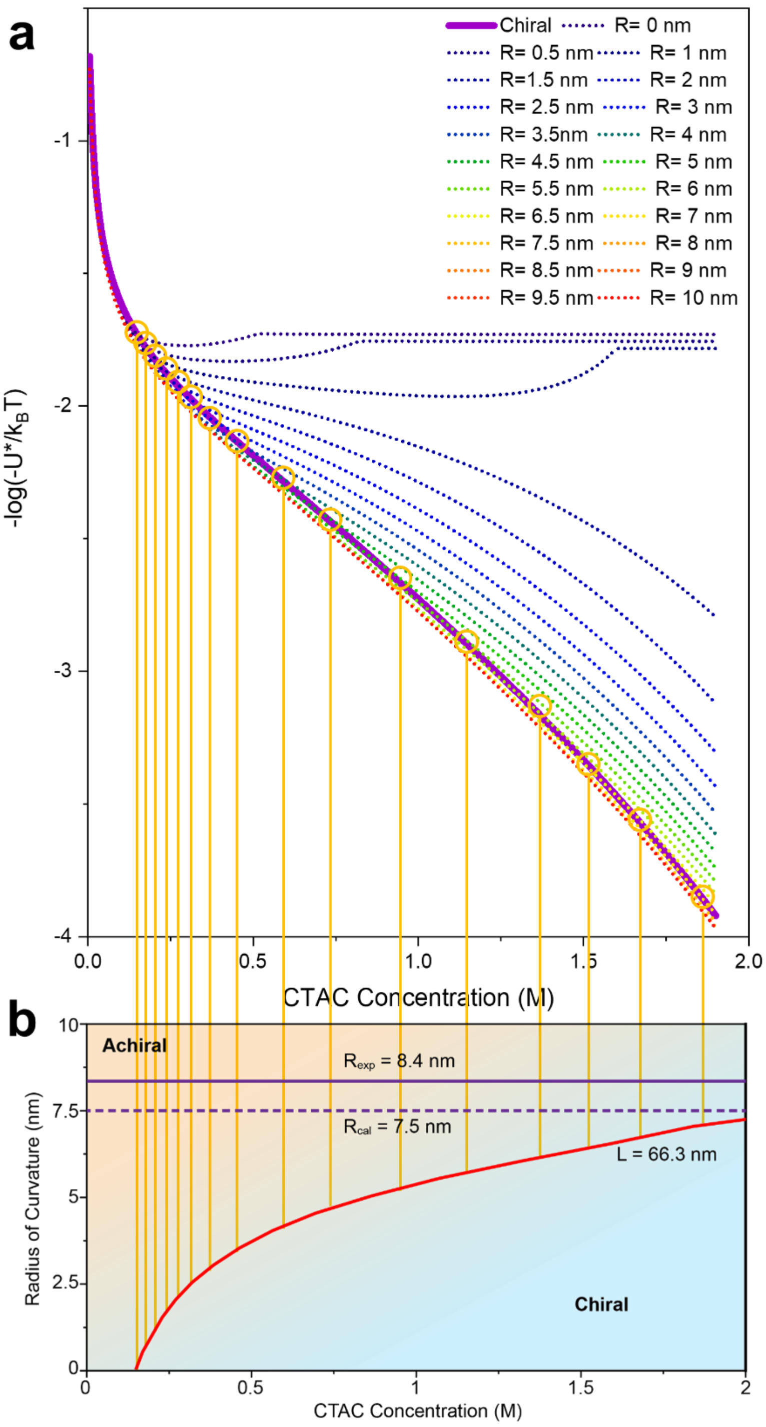

Supplementary Figure 29. a) Logarithm of net interaction energy (U_{tot}*) vs. CTAC concentration for tetrahedra (L = 66.3 nm) of different radii of curvature (R) assembled into the achiral (1) phase. For R= 7.5 nm and above, there exists no CTAC concentration at which the chiral hexagonal phase is most stable. The CTAC concentrations at which the line for the chiral phase intersects each of the radius-dependent achiral phase lines (circled) are what define the points for the concentration-dependent phase diagram presented in the maintext Fig. 3g and Fig. S25; b) Concentration-dependent phase diagram for the specific Td size used in the majority of the data presented in the maintext (L = 66.3 nm). The predicted boundary between chiral and achiral phase stability at the endpoint of the assembly process (~ 2.0 M CTAC) can be compared to electron microscopy images of dried samples obtained experimentally. The model predicts that Td with tip radii smaller than 7.5 nm should assemble into chiral superlattices but above 7.5 nm should assemble into achiral superlattices. Although our ability to control tip radius of curvature is limited to ~ 1 nm, we experimentally observe that Td with tip radii of 7.5 nm assemble into chiral superlattices but 8.4 nm assemble into achiral superlattices (Figure 3f), showing excellent agreement with the predictions of the phase diagram.

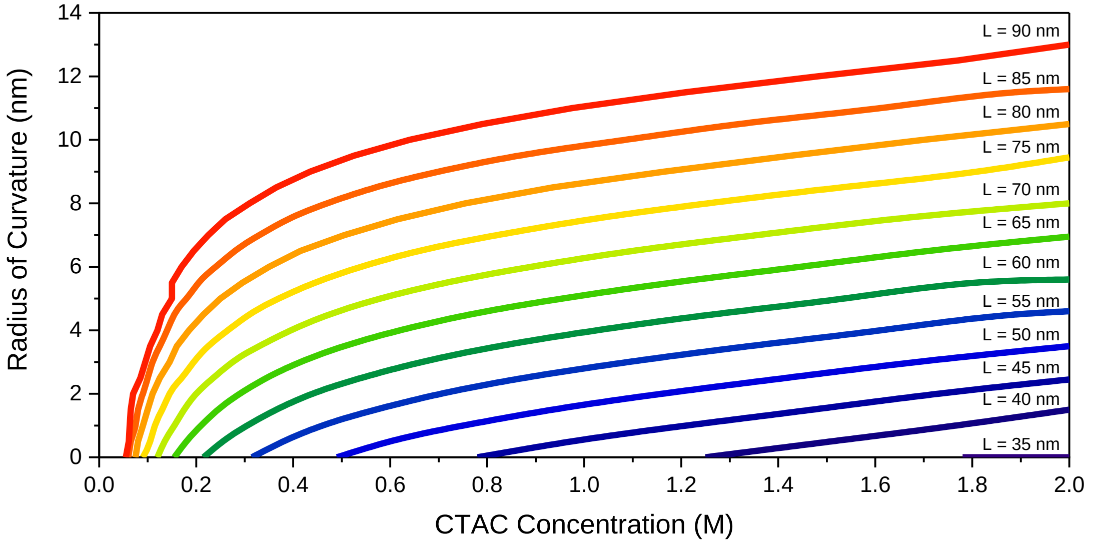

Supplementary Figure 30. Concentration-dependent phase diagram generated by comparing the relative stability of chiral (3) phase and achiral (1) phase for tetrahedra of different radii of curvature. Lines are generated from plots like that shown in Supplementary Figure 28. Regions above and below the line denote the thermodynamic stability of achiral and chiral structures, respectively.

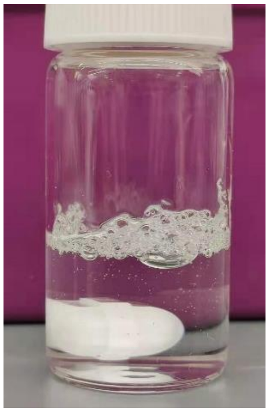

Supplementary Figure 31. Photo of 2 M CTAC solution at room temperature, indicating that high CTAC concentrations may be reached during the solvent evaporation based assembly of Td particles.

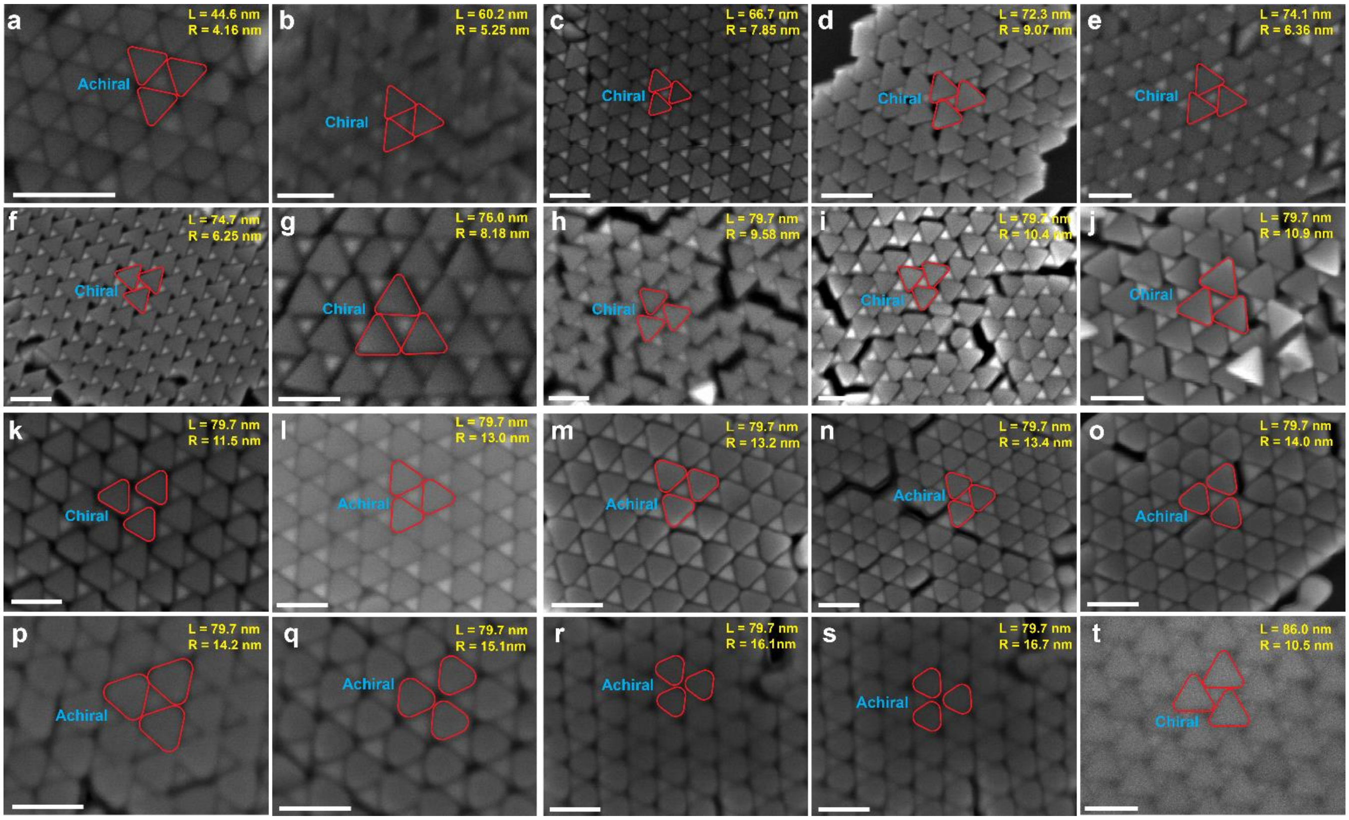

Supplementary Figure 32. SEM image compilation of multiple Td assemblies with particle size (L, a-g, t) and radius of curvature (R, b-d and h-s) written in yellow and superlattice symmetry written in blue. All scale bars are 100 nm. These data constitute the experimental points included in the phase diagram in Fig. 3h. As predicted by the theoretical model (Figure 3g, Supplementary Figure 28 and 29), small Td particles (L = 44.6 nm) can have relatively sharp tips (R = 4.16 nm) and still prefer to crystallize into the achiral phase (Supplementary Figure 31a) while large Td particles (L = 79.7 nm) with blunt tips (R = 11.5 nm) are nonetheless able to crystallize into the chiral phase (Supplementary Figure 31k). This is a consequence of the Td size-dependence of d_{min}, which is the steric threshold driving the transition from achiral to chiral superlattices via rotation (see maintext). All scale bars are 500 nm.

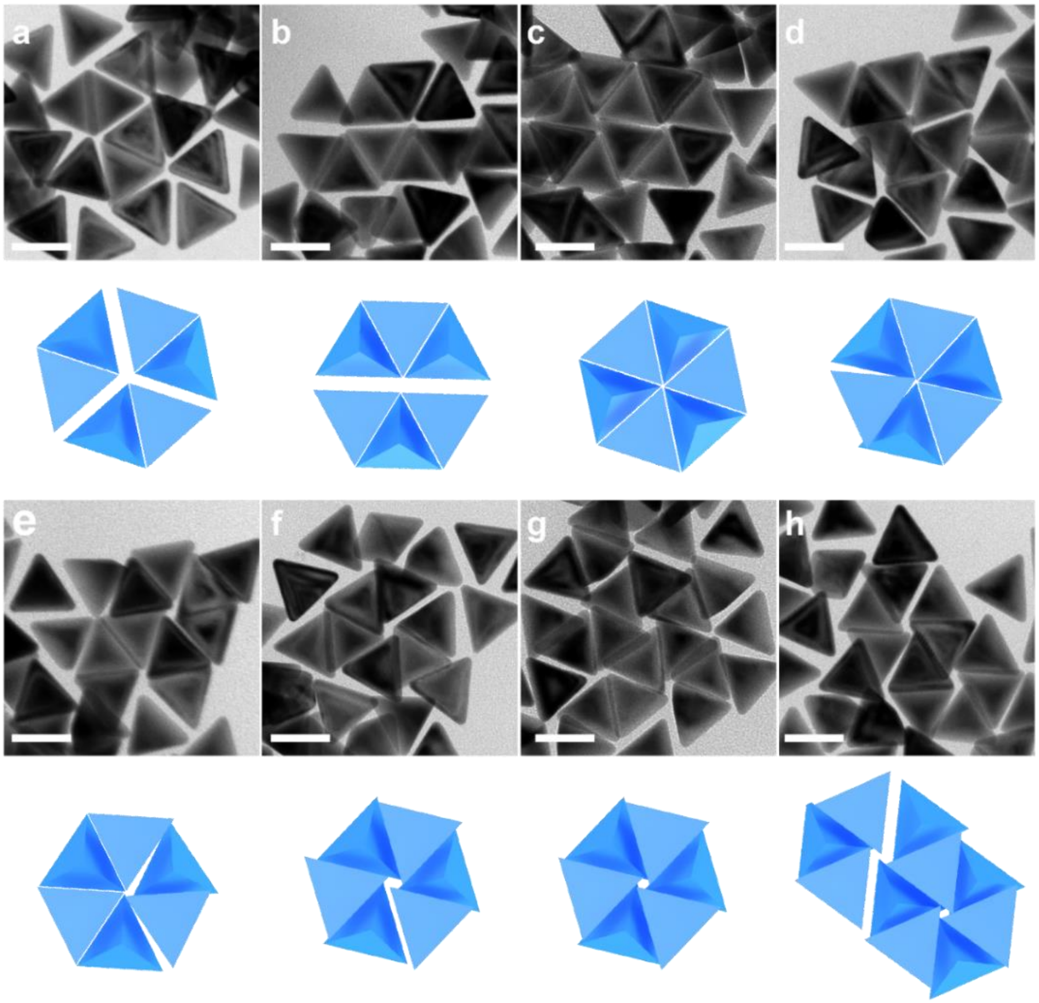

Supplementary Figure 33. TEM images collected from Au Td NPs in the middle of the solvent evaporation assembly process. Samples were prepared by quickly drying solutions on a TEM grid to trap intermediate states. Several structures (a-h) with local order suggest that the chiral (3) phase begins as the achiral (1) structure. All scale bars are 50 nm.

Supplementary References:

1. Park, K.; Koerner, H.; Vaia, R. A. Depletion-induced shape and size selection of gold nanoparticles. *Nano Lett.* **10**, 1433–1439 (2010).
2. Scarabelli, L.; Coronado-Puchau, M.; Giner-Casares, J. J.; Langer, J.; Liz-Marzán, L. M. Monodisperse gold nanotriangles: size control, large-scale self-assembly, and performance in surface-enhanced Raman scattering. *ACS Nano.* **8**, 5833-5842 (2014).
3. Lee, J.-H.; Gibson, K. J.; Chen, G.; Weizmann, Y. Bipyramid-templated synthesis of monodisperse anisotropic gold nanocrystals. *Nat. Commun.* **6**, 7571 (2015).
